# Transvenous Implantable Cardioverter‐Defibrillator (ICD) Lead Performance: A Meta‐Analysis of Observational Studies

**DOI:** 10.1161/JAHA.115.002418

**Published:** 2015-10-30

**Authors:** Rui Providência, Daniel B. Kramer, Dominic Pimenta, Girish G. Babu, Laura A. Hatfield, Adam Ioannou, Jan Novak, Robert G. Hauser, Pier D. Lambiase

**Affiliations:** ^1^ The Heart HospitalUniversity College of London Hospitals NHS TrustLondonUnited Kingdom; ^2^ Cardiac ElectrophysiologyBeth Israel Deaconess Medical CenterHarvard Medical SchoolBostonMA; ^3^ Department of Health Care PolicyHarvard Medical SchoolBostonMA; ^4^ University College of London Hospitals NHS TrustLondonUnited Kingdom; ^5^ University College of LondonUnited Kingdom; ^6^ Solothurner Spitaeler AGSolothurnSwitzerland; ^7^ Minneapolis Heart Institute FoundationMinneapolisMN; ^8^ Barts Heart CentreBarts Health NHS TrustLondonUnited Kingdom

**Keywords:** adverse event, complications, failure, fracture, implantable cardioverter‐defibrillator

## Abstract

**Background:**

Despite the widespread use of implantable cardioverter‐defibrillators (ICDs) in clinical practice, concerns exist regarding ICD lead durability. The performance of specific lead designs and factors determining this in large populations need clarification.

**Methods and Results:**

The Medline, Embase, and Cochrane Collaboration databases were searched for studies including ≥2 of the most commonly implanted leads. The Mantel‐Haenszel random‐effects model was used. Seventeen studies were selected, including a total of 49 871 patients—5538 implanted with Durata (St. Jude Medical Inc), 10 605 with Endotak Reliance (Boston Scientific), 16 119 with Sprint Quattro (Medtronic Corp), 11 709 with Sprint Fidelis (Medtronic Corp), and 5900 with Riata (St. Jude Medical Inc)—with follow‐up of 136 509 lead‐years. Although the Durata lead presented a numerically higher rate, no statistically significant differences in the mean incidence of lead failure (0.29%–0.45% per year) were observed in comparison of the 3 nonrecalled leads. A higher event rate was documented with the Riata (1.0% per‐year increase) and Sprint Fidelis (>2.0% per‐year increase) leads compared with nonrecalled leads. An indication of increased incidence of Durata lead failure versus Sprint Quattro and Endotak Reliance leads was observed in 1 of 3 included studies, allowing for comparison of purely electrical lead failure, but this requires further evaluation.

**Conclusions:**

Endotak Reliance (8F), Sprint Quattro (8F), and Durata (7F) leads displayed low annual incidence of failure; however, long‐term follow‐up data are still scarce. More data are needed to clarify the performance and safety of the Durata lead.

## Introduction

The use of implantable cardioverter‐defibrillators (ICDs) has expanded globally, with millions of patients implanted worldwide over the past 35 years.^1^, ^2^ Despite the strong evidence supporting the use of these devices to save lives,^3^, ^4^, ^5^, ^6^, ^7^, ^8^, ^9^, ^10^ concerns exist regarding the ICD lead, the weakest link of the system hardware.^11^

In the past decade, 2 ICD leads—Riata (St. Jude Medical Inc) and Sprint Fidelis (Medtronic Corp)—were recalled due to lead failure.^12^, ^13^ Despite no further implants performed with these leads, ≈495 000 patients received them (227 000 recalled Riata leads^14^ and 268 000 Sprint Fidelis leads^15^) worldwide. These lower caliber (<8‐French [<8F]) leads were thought to be advantageous (easier to implant and more likely to reduce the risk of subclavian vein thrombosis) before problems were identified.

Endotak Reliance (Boston Scientific), Sprint Quattro (Medtronic Corp), and the new 7F lead, Durata (St. Jude Medical Inc), have been the most commonly implanted ICD lead families in recent years.^16^ Nevertheless, despite their safer reputations, failures are inevitably reported. Recent studies suggested that thinner leads (<8F) may be associated with higher likelihood of lead failure.^17^ It remains to be explained whether this problem is simply due to lead caliber alone (and concomitantly more insulating material and separation between the lumens in thicker leads) or specific design deficiencies in the 2 families of leads with previously known issues (Riata and Sprint Fidelis).

Knowledge of the overall head‐to‐head performance of the most frequently used ICD lead families would be of interest not only for the referring or implanting cardiological community but also for patients with an indication for this lifesaving therapy. Unfortunately, all studies assessing performance differences among currently implanted nonrecalled leads have been underpowered to identify significant differences. A meta‐analysis of all existing data on ICD leads provides the best opportunity to address these knowledge gaps.

## Methods

### Study Selection

We performed a search in the Medline, Embase, and Cochrane Collaboration databases (from inception to July 7, 2014) using the following search string: “implantable cardioverter defibrillator” AND (“lead failure” OR “lead fracture”).

Reference lists of all accessed full‐text articles were further searched for sources of potentially relevant information. Ongoing studies assessing ICD lead failure were searched on ClinicalTrials.gov, and experts in the field were contacted to ensure that all important studies had been included. Authors of full‐text papers and congress abstracts were also contacted by e‐mail to retrieve additional information.

The PICO (population, intervention, comparison, outcome) approach was used for conducting the meta‐analysis.^18^ The population of interest included recipients of 1 of the 5 most used transvenous ICD lead families in the previous 14 years (Endotak Reliance, Sprint Quattro, Sprint Fidelis, Riata, and Durata), and the intervention was ICD implant. Comparisons were performed between the different lead families. The outcome was lead failure.

Lead failure was defined as a lead not performing according to its expected function, presenting a structural (externalization of conductors, insulation defect, or fracture) or electrical malfunction. To be considered a lead failure, the lead did not necessarily need to be extracted. Studies in which lead failure was defined exclusively on the basis of cardiac perforation and/or dislodgement were not considered eligible for inclusion.

To be included, studies needed to provide information about at least 2 of the prespecified ICD lead families, including information about all patients implanted with those leads instead of only those with failed leads. Studies including only series of patients with failed leads or collections of lead failure reports from online sources (eg, US Food and Drug Administration's Manufacturer and User Facility Device Experience [MAUDE] database) were not considered eligible.

Besides the previously mentioned exclusion criteria, studies providing no information regarding follow‐up duration and number of events for each lead family (i.e., lead failure incidence could not be ascertained) were deemed unsuitable for inclusion. Data that were from industry and published in non–peer‐reviewed sources were considered inappropriate for inclusion.

To ensure that all trials met the prespecified inclusion criteria, search results were reviewed by 3 investigators (R.P., D.P., and G.B.), who needed to reach consensus on study selection.

### Data Extraction

Data extraction and presentation for the preparation of this meta‐analysis followed Preferred Reporting Items for Systematic Reviews and Meta‐Analyses recommendations.^18^ From each study, we retrieved study design, study population characteristics (age range, sex, and type of ICD device), follow‐up duration, definition of lead failure, and lead information. Type, total number of used and failed leads for each lead family, and respective lead failure rate and incidence were collected. Included ICD lead models in this analysis were grouped into the following 5 families: Sprint Quattro (8.2F models: 6944, 6947; 8.6F model: 6935), Endotak Reliance (8.2F models: 0127, 0128, 0129, 0137, 0138, 0139, 0147, 0148, 0149, 0157, 0158, 0159, 0160, 0161, 0162, 0164, 0165, 0166, 0167, 0170, 0171, 0172, 0174, 0175, 0176, 0177, 0180, 0181, 0182, 0183, 0184, 0185, 0186, 0187), Durata (6.8F models: 7120, 7121, 7122, 7170, 7171), Riata (7.6F model: 1582; 6.7F models: 1560, 1561, 1562, 1570, 1571, 1572, 1580, 1581, 1590, 1591, 1592; 6.3F: 7000, 7001, 7002, 7010, 7011, 7040, 7041, 7042), and Sprint Fidelis (6.7F models: 6930, 6931, 6948, 6949).

Follow‐up duration in patient‐years and incidence of lead failure were retrieved directly from each study or estimated when the necessary data (number of events and follow‐up duration for each lead) were available.

Study quality was formally evaluated by 3 reviewers (R.P., D.P., and A.I.) using the Newcastle‐Ottawa Quality Assessment Scale for Cohort Studies.^19^ Agreement among the 3 reviewers was mandatory for the final classification of studies.

### Statistical Analysis

We used the Mantel‐Haenszel random‐effects model (Review Manager [RevMan], version 5.1; Cochrane Collaboration). A random‐effects model was chosen to more precisely address different effect sizes and nonuniform variation across studies.

Comparisons of the different lead families were performed using the raw mean difference of the incidence of lead failure and respective 95% CIs. Subsequently, 3 sensitivity analyses were performed: The first compared recalled (Riata and Sprint Fidelis) and nonrecalled leads (Durata, Sprint Quattro, and Endotak Reliance); the second compared leads by caliber, with 7F (Riata, Sprint Fidelis, and Durata) versus ≥8F leads (Endotak Reliance and Sprint Quattro); and the third excluded studies that included lead fixation issues, lead dislodgment, and/or perforation as part of the definition the lead failure end point.

Statistical heterogeneity of each outcome of interest was assessed and quantified using the I^2^ statistic. The I^2^ statistic describes the percentage of total variation across studies due to heterogeneity rather than chance. Values <25%, 25% to 50%, and >50% are, by convention, respectively classified as low, moderate, and high degrees of heterogeneity.

Funnel plots and meta–regression analyses were obtained using Comprehensive Meta‐Analysis software (version 2; Biostat Inc). Funnel plots were used to evaluate the presence of publication bias and traced for comparisons including >10 studies (minimum number for ensuring the appropriateness of the method).^20^

Meta–regression analysis was performed for comparisons involving >10 studies to assess the possible association of baseline differences and modulator variables (age, rate of male participants, rate of cardiac resynchronization therapy use, and mean follow‐up duration) in the observed incidence of lead failure.

## Results

### Search Results

**Figure 1:**
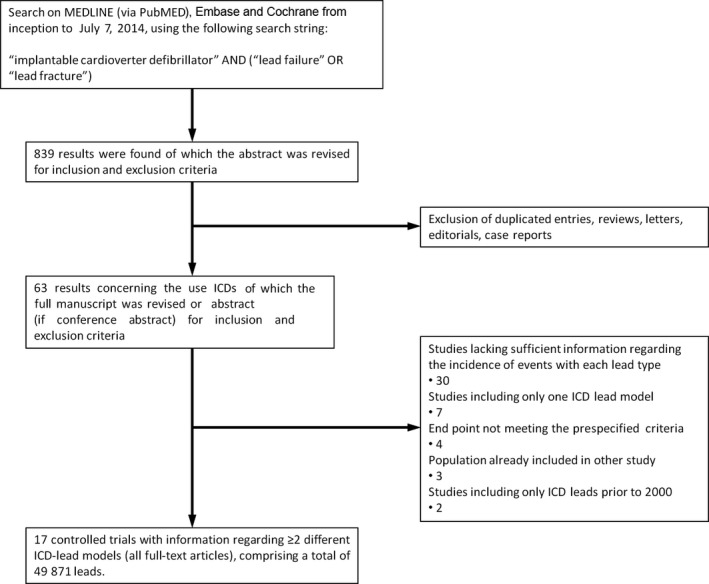
Study selection process. ICD indicates implantable cardioverter‐defibrillator.

A total of 839 entries were retrieved for analysis of titles and abstracts. Of these, 776 were excluded because they either were duplicates or were deemed unsuitable for the purpose of this meta‐analysis (editorials, letters, reviews, or case reports). The remaining 63 results were carefully screened, and after analysis of the full text (in case of journal articles), 17 studies^17^, ^21^, ^22^, ^23^, ^24^, ^25^, ^26^, ^27^, ^28^, ^29^, ^30^, ^31^, ^32^, ^33^, ^34^, ^35^, ^36^ were considered adequate for the purpose of our meta‐analysis. The selection process is illustrated in Figure 1. There was complete agreement among the investigators for the inclusion of all selected trials.

Baseline data and the design of selected trials are summarized in Table 1. Although 1 study^27^ was a subanalysis of a randomized controlled trial, no randomization was performed regarding the comparison of interest to this meta‐analysis (comparison of ≥2 families). Consequently, all studies provided observational data. Table S1 illustrates the assessment of the included studies through the Newcastle‐Ottawa scale. All included studies were assigned at least 5 stars out of a possible maximum of 8. The final population for this meta‐analysis included 49 871 leads: 5538 Durata, 10 605 Endotak Reliance, 16 119 Sprint Quattro, 11 709 Sprint Fidelis, and 5900 Riata.

**Table 1:** Main Characteristics of the Studies (n=17) Included in the Meta‐Analysis

Author, Year	Study Design Follow‐up Duration	Baseline Population	Lead Failure Definition
Kleeman et al 2007^21^	Prospective single‐center registry; follow‐up: 330 patient‐years; mean: 1.0 year	All recipients of a transvenous ICD system (first implantation); age 62.6±10 years; 79.7% male; dual‐chamber, 38.3%; CRT, 15.1%	Severe lead failure that required surgical correction. Lead failure required ≥1 of the following: (1) Oversensing unrelated to the cardiac cycle was documented; (2) lead impedance was out of normal range, and a surgical revision was suggested by the experts of the manufacturer; (3) a fracture was observed on x‐ray; (4) evidence existed of a lead failure during electrical testing
Eckstein et al 2008^22^	Retrospective multicentric (3 sites) study; follow‐up: 2426 patient‐years; median: 6.4 years	All patients implanted with transvenous ICD leads over a decade	Lead‐related problem requiring surgical revision to be corrected. Lead problems were defined as structural problems, including insulation defects and lead fractures, and functional problems, including noise or far‐field sensing, T‐wave oversensing, and others (noise resulting from contact with another lead, unstable impedance measurements, R‐wave reduction, and loss of capture)
Hauser et al 2008^23^	Retrospective multicentric (7 sites) study; follow‐up: 582 patient‐years; mean: 3.3 years	All patients with HCM receiving transvenous high‐voltage ICD leads; age 47±16 years; 67.0% male	Lead undersensed or oversensed normal cardiac electrical activity or could not provide effective electrical therapy (including sensing, pacing, or defibrillation) as the result of a noniatrogenic device‐related defect in the insulation, conductor, electrode, shocking coil, fixation mechanism, or terminal pin, ring, or seal that connects the lead to the ICD pulse generator
Borleffs et al 2009^24^	Retrospective single‐center study; follow‐up: 4330 patient‐years; mean: 2.4 years	All recipients of ICD leads; age 61±13 years; 80% male; dual chamber, 49%; CRT, 36%	At least 1 of the following criteria had to be met to define suspected lead failure (1 and 2) or verified lead failure (3–6): (1) loss of capture or markedly elevated thresholds; (2) loss of sensing, oversensing, or skeletal muscular stimulation; (3) a visible conductor fracture or insulation defect seen at surgery; (4) a change in lead impedance, judged to be caused by conductor or insulation failure; (5) an evident fracture seen on chest roentgenogram; or (6) manufacturer's returned product report confirming the failure
Hauser and Hayer 2009^25^	Prospective multicentric (2 sites) registry; follow‐up: 5390 patient‐years; mean: 1.9 years	All patients implanted with ICD leads	Lead undersensed or oversensed normal cardiac electrical activity or if it could not provide effective electrical therapy including sensing, pacing, or defibrillation. Displacements after the index procedure were considered a failure only if the lead had to be removed from service and was found to have a defective fixation mechanism
Morrison et al 2011^26^	Prospective multicentric (3 sites) study; follow‐up: 8421 patient‐years; mean: 3.2 years	All recipients of the prespecified ICD leads; age 64.5±14.6 years; 77.3% male; CRT, 31.4%	Lead removed from service due to an inability to meet its performance specifications or otherwise perform as intended. A lead failed if (1) it exhibited abnormal impedance; (2) it exhibited electrical noise as manifested by nonphysiological signals on the electrogram or by pulse generator diagnostic data suggesting rapid oversensing, for example, nonphysiological short intervals and/or recurrent nonsustained ventricular tachycardia with intervals usually <220 ms; or (3) it could not sense R waves and/or provide effective electrical therapy due to apparent structural defect such as a conductor fracture or insulation breach
Parkash et al 2012^27^	Randomized controlled trial (RAFT) subanalysis; follow‐up: 5808 patient‐years; mean: 3.3 years	All participants in the trial; age 66.1±9.4 years; 83% male; CRT, 49.7%	If 2 of the following: (1) impedance rise (>50% or 500 Ω in 1 week), (2) short interval count >10 times per day or 300 times per month, and (3) inappropriate shock because of noise recorded on the electrogram
Sung et al 2012^28^	Prospective multicentric (150 sites) US Department of Veteran Affairs registry; follow‐up: 51 592 patient‐years; mean: 3.5 years	All patients implanted with the prespecified leads	If 1 of the following was met: (1) presence of nonphysiological noise not due to external interference such as electromagnetic interference; (2) rise in pace/sense (p/s) conductor impedance to >2000 Ω usually from baseline impedance <1000 Ω or >2× rise in stable baseline impedance; (3) drop in p/s conductor impedance to less than half of the previously stable baseline value or to impedance <200 Ω from baseline impedance >300 Ω; (4) change in superior vena cava or high‐voltage coil impedance to >200 or <25 Ω; and (5) rise in capture threshold to >2 times the previously stable value
Abdelhadi et al 2013^29^	Retrospective multicentric (7 sites) study; follow‐up: 9509 patient‐years; mean: 3.5 years	Adults implanted with the prespecified ICD leads; age 65±13 years; 76.9% male	If the lead did not perform according to its specifications or otherwise function as intended, including electrical malfunction and externalized conductors
Ellenbogen et al 2013^30^	Retrospective multicentric study; follow‐up: 11 424 patient‐years; mean: 0.9 year	All patients with a Medtronic ICD with LIA enabled; age 66.3±14.3 years; 73.1% male; dual chamber, 38.0%; CRT, 45.6%	Lead‐system event, 1 of the following: lead failure, connector issue, dislodgement, perforation, and lead–lead interaction. Lead‐failure was defined based on electrogram tracings, sensing integrity counters, RR intervals, and lead impedance trends
Fazal et al 2013^31^	Retrospective single‐center study; follow‐up: 984 patient‐years; mean: 4.5 years	All patients implanted with the prespecified ICD leads; age 64.4±18.5 years; 83.8% male; dual chamber, 31.0%; CRT, 33.3%	Any of the following: (1) sudden increase in pacing or defibrillation impedance from baseline without alternative explanation; (2) frequent short V–V intervals implying fracture of the conductor, contact between the 2 components generating electrical “noise” (sensing artifact); (3) delivery of inappropriate shock(s) as a consequence of interpretation of these sensing artifacts as ventricular arrhythmia; and (4) failure of effective electrical therapy including sensing, pacing, or defibrillation
Rordorf et al 2013^17^	Retrospective single‐center study; follow‐up: 4515 patient‐years; mean: 5.2 years	All patients implanted with transvenous ICD leads; age 57±13 years; 83% male; dual chamber, 7%; CRT, 29%	Sudden change (≥50% compared with chronic values) in long‐term pacing and high‐voltage impedance and/or electrical noise artifacts from rapid, nonphysiological make‐break potentials recorded on the sensing channel. Lead dislocation or perforation or oversensing of noncardiac potentials, not considered indicative of lead integrity failure (ie, electromagnetic interferences), were not considered in this analysis
Verlato et al 2013^32^	Prospective multicentric (12 sites) registry; follow‐up: 2336 patient‐years; mean: 2.4 years	All patients implanted with the prespecified leads; age 67±10 years; 83.5% male; dual chamber, 17.1%; CRT, 67.3%	In the absence of noise‐induced shocks, lead failure evidence was derived from the analysis of signals such as a significant decrease or increase in lead impedance; presence of false nonsustained ventricular tachycardia episodes; significant increase in the sensing integrity counter, which counts the number of sensed, nonphysiological short ventricular intervals near the ICD blanking period; oversensing or undersensing of the normal cardiac electrical activity, as registered on the right ventricular channel; or any change in electrical parameters which may prevent detection or interruption of potentially lethal arrhythmias
Liu et al 2014^33^	Retrospective single‐center study; follow‐up: 19 237 patient‐years; mean: 3.6 years	All ICD recipients; age 68±13 years; 76.9% male; CRT, 39.1%	Electrical malfunction resulting in lead extraction or replacement with a new ICD lead was defined as abnormal pace sense or high‐voltage impedance values, decrease in R‐wave amplitude, increase in pacing threshold necessitating lead replacement; or the presence of electrical noise leading to inappropriate ICD therapy. Leads demonstrating mechanical failure (eg, cable externalization in Riata leads) with normal electrical function were not classified as lead failures in the present study
Vollman et al 2014^34^	Retrospective single‐center study; follow‐up: −2774 patient‐years; mean: 3.7 years	All patients implanted with the prespecified leads; age 62.3±14.9 years; 81.0% male; CRT, 30.1%	A lead failed if ≥1 of the following criteria applied: (1) sudden rise in long‐term pacing or high‐voltage impedance; (2) electrical noise artifacts as manifested by nonphysiological signals on the electrogram or by device diagnostics; (3) failure to sense R‐waves or ineffective electrical therapy due to an apparent structural lead defect
Yanagisawa et al 2014^35^	Retrospective single‐center study; follow‐up: 779 patient‐years; mean: 3.6 years	All patients implanted with the prespecified ICD leads; age 60.2±15.2 years; 73.4% male; CRT, 38.8%	Nonphysiological high‐rate sensing; sudden change of sense and pace, coil impedance out of normal limits, or inappropriate shock due to sensing of electrical noise artifacts were suggestive of lead failure
Kramer et al 2015^36^	Retrospective multicentric (4 sites) study; follow‐up: 8797 patient‐years; median: 3.2 years	All patients implanted with the prespecified leads; age 65 years (IQR 55–74 years); 74% male	Failure definition included ≥1 of the following: (1) impedance outside the labeled normal range for that model; (2) electrical noise manifest as nonphysiological signals on the electrogram or as pulse generator diagnostic data suggesting rapid oversensing, eg, nonphysiological short intervals and/or recurrent nonsustained ventricular tachycardia with intervals usually <220 ms; (3) increase in pacing threshold or decline in R‐wave amplitude necessitating lead replacement; (4) inability to provide effective therapy due to a lead defect; (5) externalized conductor that breached the outer insulation and appeared outside the lead body on fluoroscopy or radiography; and (6) lead dislodgement, except simple dislodgments without an identified fixation mechanism defect. Functional abnormalities, including exit block and physiological oversensing in an electrically intact lead, were not considered as failures

CRT indicates cardiac resynchronization therapy; HCM, hypertrophic cardiomyopathy; ICD, implantable cardioverter‐defibrillator; IQR, interquartile range; LIA, lead integrity alert; RAFT, Resynchronization/Defibrillation for Ambulatory Heart Failure trial.

### Performance of the Different Lead Families

During a total follow‐up of 136 509 lead‐years, 1265 lead failures reportedly occurred, accounting for an incidence of 0.93 event per 100 lead‐years (95% CI 0.88–0.98).

Longer follow‐up was available regarding the Sprint Quattro (49 689 lead‐years), Sprint Fidelis (35 300 lead‐years), and Endotak Reliance (27 479 lead‐years) lead families. The Durata family had shorter follow‐up (6716 lead‐years). Mean follow‐up in the included studies was usually ≈2 to 3 years, shown in 13 of the17 selected studies^23^, ^24^, ^25^, ^26^, ^27^, ^28^, ^29^, ^32^, ^33^, ^34^, ^35^, ^36^ (Table 1). None of the included studies presented >6 years of median follow‐up; the longest was 6.4 years (95% CI 6.0–6.6).^22^

**Figure 2:**
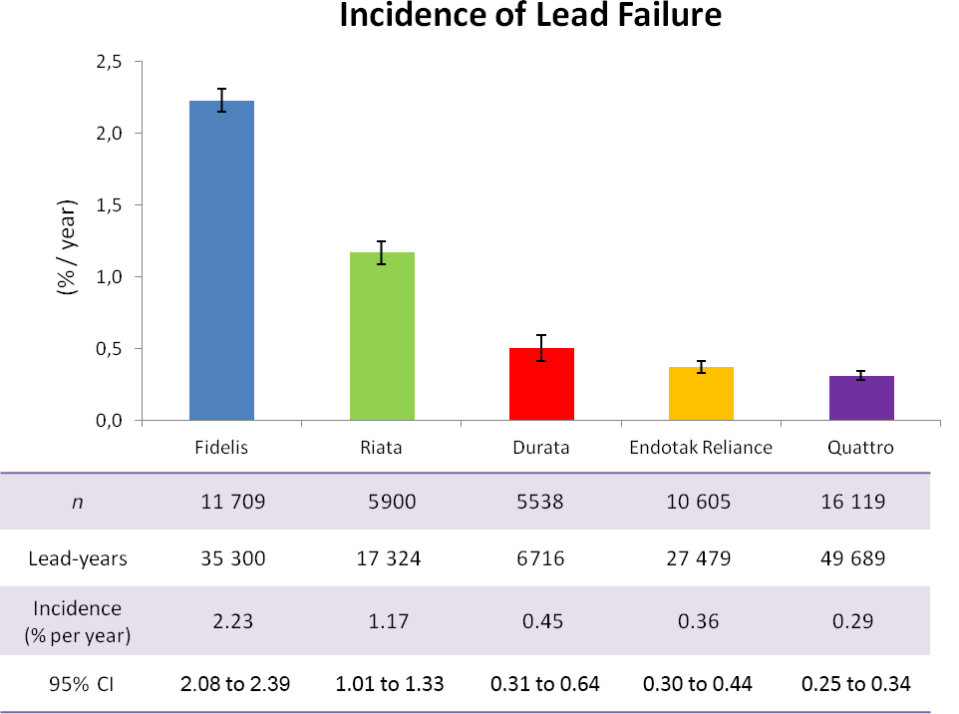
Incidence of lead failure.

The incidence of lead failure in the selected articles is shown in Table 2. Pooling of all data showed low annual incidence of lead failure in all 3 nonrecalled lead families, with values ranging between 0.29% and 0.45% per year (Sprint Quattro 0.29% [95% CI 0.25–0.34], Endotak Reliance 0.36% [95% CI 0.30–0.44], Durata 0.45% [95% CI 0.31–0.64]) (Figure 2). In recalled leads, the incidence of failure increased to 1.17% per year with the Riata lead and 2.23% per year with Sprint Fidelis.

**Table 2:** Main Events in the Studies Included in the Meta‐Analysis

Study	Durata			Endotak Reliance			Fidelis			Quattro			Riata		
	Failure Rate	Lead‐Years	Incidence 95% CI	Failure Rate	Lead‐Years	Incidence 95% CI	Failure Rate	Lead‐Years	Incidence 95% CI	Failure Rate	Lead‐Years	Incidence 95% CI	Failure Rate	Lead‐Years	Incidence (95% CI)
Kleeman 2007^21^	—	—	—	0/1, 0%	6.6	0 (0–36.93)	0/1, 0%	0.27	0 (0–93.43)	0/139, 0%	155.7	0 (0–7.25)	13/199, 6.53%	167.2	7.78 (4.60–12.85)
Eckstein 2008^22^	—	—	—	1/159, 0.63%	1017.6	0.10 (0.02–0.55)	—	—	—	3/103, 2.91%	659.2	0.46 (0.15–1.33)	0/42, 0%	268.8	0 (0–1.41)
Hauser 2008^23^	—	—	—	2/89, 2.25%	247.3	0.81 (0.22–2.90)	4/69, 5.80%	102.1	3.92 (1.53–9.64)	0/70, 0%	190.9	0 (0–1.97)	1/35, 2.86%	40.8	2.45 (0.43–12.65)
Borleffs 2009^24^	—	—	—	15/911, 1.65%	1954.3	0.77 (0.47–1.26)	16/376, 4.26%	584.1	2.74 (1.69–4.40)	20/322, 6.21%	1284.8	1.56 (1.01–2.39)	4/183, 2.19%	504.1	0.79 (0.31–2.02)
Hauser 2009^25^	—	—	—	7/607, 1.15%	1241	0.56 (0.27–1.16)	72/848, 8.49%	1923	3.74 (2.98–4.69)	11/1273, 0.86%	2013	0.55 (0.31–0.98)	1/90, 1.11%	213	0.47 (0.08–2.61)
Morrison 2011^26^	—	—	—	—	—	—	85/1030, 8.25%	2952.7	2.88 (2.33–3.55)	23/1641, 1.40%	5454.3	0.42 (0.28–0.63)	—	—	—
Parkash 2012^27^	—	—	—	—	—	—	45/818, 5.50%	2658.5	1.69 (1.27–2.26)	2/969, 0.21%	3149.25	0.06 (0.02–0.23)	—	—	—
Sung 2012^28^	—	—	—	12/2401, 0.50%	7580	0.16 (0.09–0.28)	379/5073, 7.47%	18 255	2.08 (1.88–2.30)	28/6091, 0.46%	18 748	0.15 (0.10–0.22)	47/1403, 3.35%	7039	0.67 (0.5–0.89)
Abdelhadi 2013^29^	—	—	—	—	—	—	—	—	—	23/1668, 1.38%	5304.2	0.43 (0.29–0.65)	47/1081, 4.35%	4205.1	1.12 (0.84–1.48)
Ellenbogen 2013^30^	10/4179=0.24%	3343.2	0.30 (0.16–0.55)	24/3654, 0.66%	3288.6	0.73 (0.49–1.08)	24/1556, 1.54%	2800.8	0.86 (0.58–1.27)	—	—	—	21/1944, 1.08%	1749.6	1.20 (0.79–1.83)
Fazal 2013^31^	—	—	—	—	—	—	13/113, 11.50%	499.5	2.61 (1.53–4.40)	—	—	—	13/106, 12.26%	484.4	2.62 (1.57–4.54)
Rordorf 2013^17^	0/86=0%	157.7	0 (0–2.38)	7/204, 3.43%	663	1.06 (0.51–2.16)	29/190, 15.26%	680.2	4.8 (2.98–6.06)	0/130, 0%	650	0 (0–0.90)	16/182, 8.79%	606.6	2.64 (1.63–4.25)
Verlato 2013^32^	—	—	—	—	—	—	27/508, 5.31%	1437.6	1.88 (1.29–2.72)	2/468, 0.43%	898.6	0.22 (0.06–0.81)	—	—	—
Liu 2014^33^	18/828=2.17%	1904.4	0.95 (0.60–1.49)	26/2190, 1.32%	10 074	0.26 (0.18–0.38)	47/623, 7.54%	1682.1	2.79 (2.11–3.70)	11/1020, 1.08%	3570	0.31 (0.17–0.55)	38/627, 6.06%	2006.4	1.89 (1.38–2.59)
Vollmann 2014^34^	—	—	—	—	—	—	33/429, 7.69%	1394.3	2.37 (1.69–3.31)	5/276, 1.81%	1380	0.36 (0.15–0.85)	—	—	—
Yanagisawa 2014^35^	—	—	—	—	—	—	14/75, 18.67%	360	3.89 (2.33–6.42)	0/131, 0%	379.9	0 (0–1.00)	1/8, 12.5%	39.2	2.55 (0.45–13.12)
Kramer 2015^36^	2/445=0.45%	1311	0.15 (0.04–0.55)	6/389, 1.54%	1407	0.43 (0.20–0.93)	—	—	—	17/1818, 0.94%	6079	0.28 (0.17–0.45)	—	—	—
Pooled Data	30/5538=0.54%	6716.3	0.45 (0.31–0.64)	100/10 605, 0.94%	27 479.3	0.36 (0.30–0.44)	788/11 709, 6.73%	35 300.2	2.23 (2.08–2.39)	145/16 119, 0.90%	49 689.3	0.29 (0.25–0.34)	202/5900, 3.42%	17 323.6	1.17 (1.02–1.34)

Failure rate in percentage of total leads of each model; incidence in percentage per year.

Data analysis restricted to only head‐to‐head comparisons of the different leads is summarized in Table 3 (forest plots are shown in Figure S11). No significant differences in the mean incidence of lead failure were observed in comparisons between nonrecalled leads (mean difference: Endotak Reliance versus Durata 0.14% [95% CI −0.48 to 0.77], *P*=0.66; Sprint Quattro versus Durata −0.25% [95% CI −0.75 to 0.24], *P*=0.31; Endotak Reliance versus Sprint Quattro 0.01% [95% CI −0.17 to 0.19], *P*=0.91). Moreover, all comparisons of 7F recalled leads versus nonrecalled leads led to significant differences confirming the higher event rates, showing increases of 1.0% per year with the Riata lead and >2.0% per year with Sprint Fidelis. Comparison of the 2 recalled leads yielded a significant 1.24% annual increase in failure with the Sprint Fidelis lead.

**Table 3:** Results of the Head‐to‐Head Comparison of the 5 Lead Families (Differences in Mean Incidence and Study Heterogeneity)

Durata	Endotak Reliance	Fidelis	Sprint Quattro	Riata
Durata	0.14 (−0.48 to 0.77); *P*=0.66; I^2^=83%	−3.12 (−5.77 to −0.47); *P*=0.02; I^2^=73%	−0.25 (−0.75 to 0.24); *P*=0.31; I^2^=55%	−1.25 (−2.01 to −0.49); *P*=0.001; I^2^=61%
	Endotak Reliance	−2.20 (−3.03 to −1.36); *P*<0.001; I^2^=89%	0.01 (−0.17 to 0.19); *P*=0.91; I^2^=36%	−0.57 (−1.01 to −0.13); *P*=0.01; I^2^=58%
		Fidelis	−2.29 (−2.71 to −1.88); *P*<0.001; I^2^=63%	−1.24 (−2.08 to −0.40); *P*=0.004; I^2^=75%
			Sprint Quattro	−0.73 (−1.29 to −0.16); *P*=0.01; I^2^=78%
				Riata

The I^2^ values showed moderate heterogeneity in the observed results of the Endotak Reliance versus Sprint Quattro comparison. All remaining comparisons displayed high heterogeneity values.

### Sensitivity Analyses

**Figure 3:**
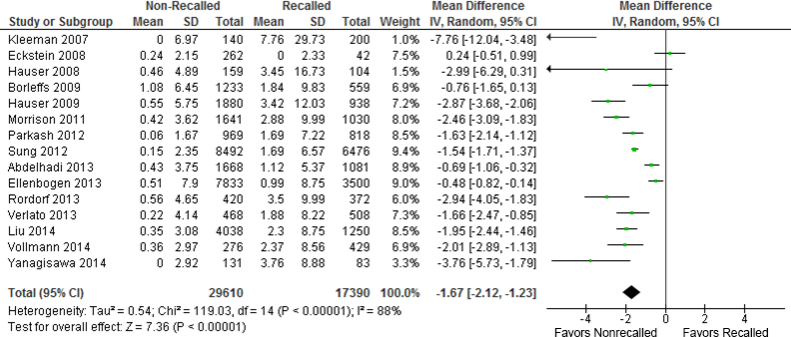
Sensitivity analysis of recalled versus nonrecalled leads.

In the sensitivity analysis of recalled versus nonrecalled leads (Figure 3), the forest plot showed the clear benefit of the latter group of leads, with 1.67% lower incidence of lead complications per year. Despite the observed high heterogeneity (relating mostly to the magnitude of the difference), the forest plot clearly showed benefit in favor of nonrecalled leads.

**Figure 4:**
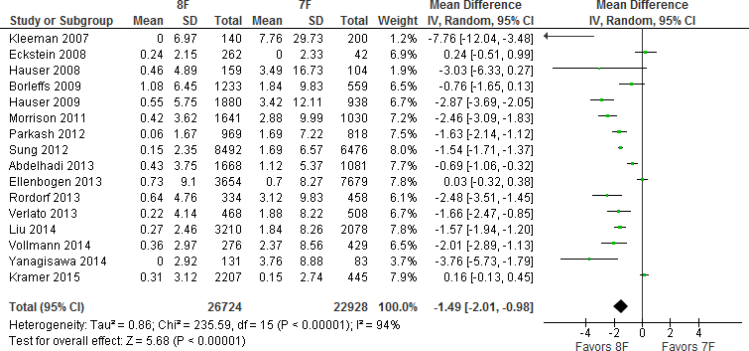
Sensitivity analysis of 7‐ versus ≥8‐French leads.

When comparing lead caliber (Figure 4), 1.49% more events per year were observed with 7F leads; however, when Sprint Fidelis and Riata lead families were excluded from analysis, comparisons of Durata versus Endotak Reliance and Sprint Quattro did not confirm significant increases in lead failure with the most recent 7F lead family (Figures S1 through S3).

Two studies^25^, ^30^ were excluded from the last sensitivity analyses because they included some cases of purely mechanical lead failure in the end point definition. Careful revision of the raw data from a third study^36^ with possibility of mechanical lead failure in its end point allowed us to keep the study in this sensitivity analysis because no purely mechanical lead failures occurred.

Head‐to‐head comparisons of the different lead families are presented in Table S2 and in Figures S1‐S10. The results of these comparisons were similar to those in the main analysis.

The funnel plot analyses excluded the presence of selection bias among the included studies (all studies are located below the 95% CIs of the plot) (Figure S11).

**Figure 5:**
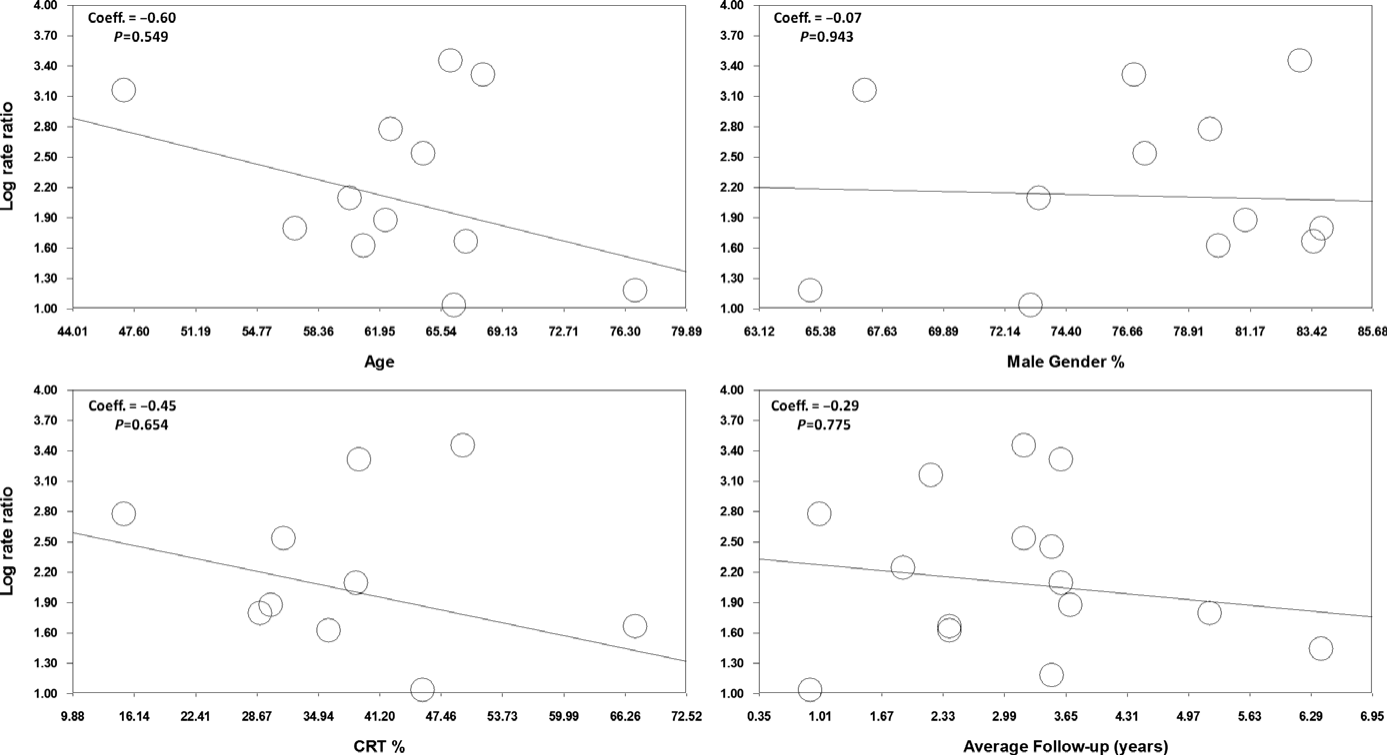
Meta‐regression analysis of head‐to‐head comparisons of the different lead families. Coeff. indicates coefficient; CRT, cardiac resynchronization therapy.

The results of meta–regression analysis for these 2 analyses confirmed that none of the assessed modulator variables (age, rate of male participants, mean follow‐up duration, and rate of cardiac resynchronization therapy use, which differed slightly among studies) displayed a significant association with lead failure incidence in comparisons of recalled versus nonrecalled leads (Figure 5) and 7F versus ≥8F leads (Figure S12).

## Discussion

These data indicate that the risk of lead failure is non‐negligible for all assessed lead families (overall 0.93 event per 100 lead‐years; ie, almost 10% at 10 years, ranging from 2.9% to 4.5% in the nonrecalled lead families) and seems to depend mainly on lead family design rather than on lead diameter. The annual incidence of lead failure among the 2 standard‐size lead families (Sprint Quattro and Endotak Reliance) was very low and was comparable (no significant differences were found, and study results had low heterogeneity). The thinner 7F Durata lead, however, also seemed to display a similar durability profile in the main analysis, despite the slightly shorter follow‐up duration and lower number of patients (a result of its most recent release date). Consequently, not all 7F leads seem to be associated with increased risk of failure at first glance. The significant increase in the incidence of failure that was observed when comparing all 7F and ≥8F leads seemed to be driven by the negative outcomes of the recalled leads; however, we believe that reassurance regarding the Durata lead may still be premature. Although it did not perform unfavorably in the head‐to‐head comparisons with the Sprint Quattro or Endotak Reliance leads, only 3 studies are available to compare purely electrical failure of the Durata lead with the Sprint Quattro and Endotak Reliance leads, and in 1 of those studies,^33^ both comparisons were unfavorable (Figures S2 and S3, Table S2). Another note of caution comes from the numerically higher (although not statistically significant) failure rate of the Durata lead (0.45% per year) compared with the Endotak Reliance (0.36% per year) and Sprint Quattro (0.29% per year) lead families.

Concerns exist regarding a possible exponential increase in failure rate with time; however, meta–regression results confirmed that variations in follow‐up duration were not associated with increases in failure rate in the comparisons of recalled versus nonrecalled leads (Figure 5) and 7F versus 8F leads, which may be reassuring for the time being (Figure S12). Definite reassurance regarding the Durata lead family will be possible only as more data with longer follow‐up duration become available.

**Figure 6:**
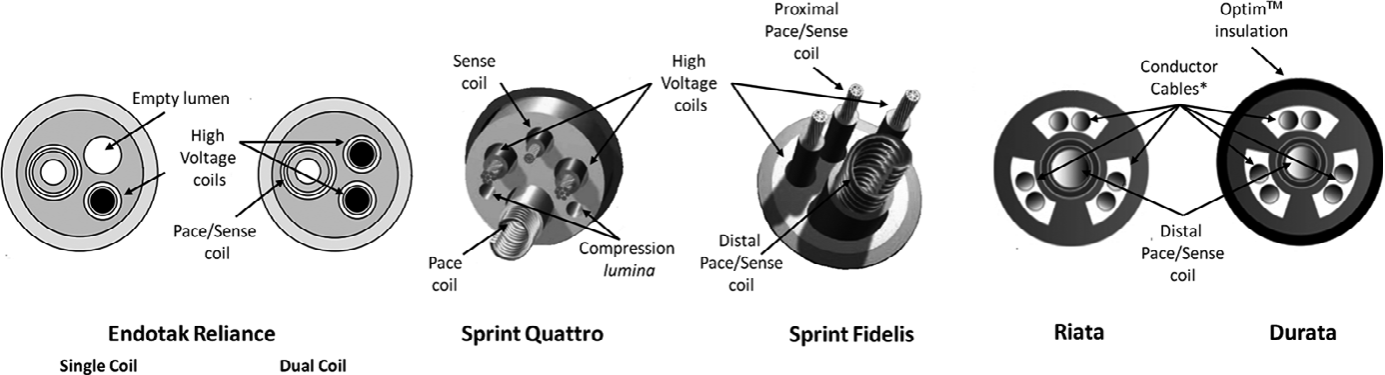
Construction and materials of the different lead families.

Our study highlights the importance of engineering design and preclinical and clinical testing before wide introduction into the market for a life‐saving device intended to be permanent and durable in a hostile biological environment. These considerations are critical as major manufacturers continue to market new lead models—including DF‐4 connectors, VDD ICD leads, and systems designed for compatibility with magnetic resonance imaging—with limited premarket clinical testing. What has been learned so far? Sprint Fidelis is a 6.7F lead with silicone insulation and a polyurethane outer coating with an asymmetrical design in cross‐section. Failure originated in a vulnerable pace‐sense conductor that was prone to fracture (occurring 90% of the time in the conductor to the ring electrode), causing oversensing of electrical artifacts, undersensing of ventricular depolarization, and/or loss of ventricular capture.^37^ This lead resembled construction and materials with the previously launched and thicker Sprint Quattro model (Figure 6). Besides displaying a greater distance between the conductors and the outer limit of the lead due to thicker layers of silicone and polyurethane, the Sprint Quattro also featured 2 compression lumens that may affect its longevity.^37^ As of August 3, 2014, there were 540 512 registered Sprint Quattro ICD lead implants in the United States.^38^

The Endotak Reliance lead is asymmetrical on cross‐section and has fewer conductors, which are coated with polytetrafluoroethylene and insulated in separate lumens within the silicone rubber lead body. Finally, the lead is covered by a second outer layer of silicone.^39^ As of July 21, 2014, >658 000 leads were implanted worldwide.^40^ Lead engineers should put emphasis on the importance of lead design and its role in the 2 lead families with the longest follow‐up and the best results, Sprint Quattro and Endotak Reliance.

Issues with the Riata lead resulted from insulation abrasion, which is thought to occur as a result of repetitive motion of the ethylene tetrafluoroethylene–coated cables within the silicone‐walled lumens. This seems to exert disruptive forces leading to “inside‐out” insulation failure, frequently manifest by externalized cables.^41^

The Durata lead underwent design improvements to provide greater resistance to abrasion and protection against externalized conductors with the addition of Optim (St. Jude Medical Inc), a silicone and polyurethane copolymer 50 times more abrasion resistant than silicone. The addition of the Optim insulation layer increases insulation thickness by 50%. Major design elements of the Durata are similar to the Riata lead, with a symmetrical cross‐section in which ethylene tetrafluoroethylene–coated cables run through large lumens^42^ (Figure 6). Since its introduction in 2007, it is estimated that 404 598 Durata leads had been implanted worldwide as of May 2014.^43^

Comparison of the overall failure rate for each lead family with the results of product performance reports provided by manufacturers showed that ICD leads in the real world may be performing slightly below expectations. Lead survival probability 10 years after implant for most Endotak Reliance leads on Boston Scientific's performance report is >98% to 99%,^40^ whereas our results, with shorter duration follow‐up, showed an annual failure rate of 0.36%. This suggests that some physicians are not reporting cases of lead failure to the regulatory authorities and manufacturers, and 96% to 97% (if the annual rate of lead failure remains stable over time) may be more accurate.

Data in this meta‐analysis are unable to resolve some of the concerns regarding 7F leads, namely, their durability over time.^17^ Most studies included in the meta‐analysis had a mean follow‐up duration of 2 to 3 years, and none displayed >6 years of average follow‐up duration. Nevertheless, incidence seemed to remain constant and similar to that observed with Endotak Reliance and Sprint Quattro in the first few years. Preliminary data suggest that the Endotak Reliance and Sprint Quattro leads seem to have a stable performance over time.^44^ In the first 5 years after implant, performance of the Durata lead also seems promising^45^; however, its performance over a longer follow‐up period is still unknown. Over time, the hazard of lead fracture or conductor externalization increased exponentially with the 2 recalled 7F leads.^25^, ^46^, ^47^ Can failure rate also increase over time in a lead presenting with good initial reliability? Data are still absent regarding the Durata lead after the first 4 to 5 years and the Endotak Reliance and Sprint Quattro family leads implanted for >10 years. This matter should be addressed in future studies because more young adults with channelopathies and cardiomyopathies are being implanted with ICDs for which intravenous leads would be expected to remain in place for decades.

The experience from recalled ICD lead families in past decades shows the need for thoughtful partnership between industry and academia (including the major ICD registries and health care systems) to design, test, and monitor ICD leads in the pre‐ and postmarketing settings.^48^ A close and active surveillance system that can detect low‐frequency failures or adverse clinical events involving the most recent ICD leads, or any other marketed medical device, should be a priority. No longer should we rely on passive postmarketing surveillance strategies, which have been shown to fail to detect significant device defects before large patient populations have been exposed.^48^, ^49^ Some prospects of improvement are expected with the introduction of remote monitoring because automated resources are now available to prospectively monitor large multicenter device databases for early, low‐frequency adverse events.^48^ In addition, the implementation of a unique device identifier system to provide more accurate reporting, review, and analysis of adverse events may be of potential interest to identify and correct real‐world problems.^50^

Finally, the existence of ICD lead families that already have track records of proven performance and that can allow most patients to achieve a goal of “one lifetime, one lead” should prompt physicians to select leads based on this principle and “stop experimenting with new leads outside the context of investigational studies that require institutional review board approval and informed consent.”^49^

The new subcutaneous ICD may be way of overcoming these concerns about lead longevity in specific population subsets, especially younger patients without pacing indications or a need for antitachycardia pacing who may face decades of device prophylaxis. The subcutaneous ICD lead is potentially more resilient and much easier to extract in case of failure due to its subcutaneous location.^51^

### Limitations

Several limitations are commonly linked to the methodology of meta‐analyses, which involve cross‐study comparisons. First, because of the very low incidence of lead failure, the results obtained from methods assessing study heterogeneity (e.g., I^2^) must be interpreted with caution. Events occurring in small samples may produce large variations and overestimation of the incidence of events; however, all results show a clear mean difference with a very narrow 95% CI, which provides a clear message concerning existence or lack of differences among the different lead comparisons. Second, differences in study design may also affect the observed differences. Third, minor differences exist in the definition of lead failure among the different studies and may contribute to the observed heterogeneity. Fourth, reporting bias may have a role in these differences because studies showing pronounced differences (higher or lower failure rates) may have a greater likelihood of being reported or published; however, these results were consistent, namely, more events with recalled leads with no marked differences between the nonrecalled leads. Most of the observed differences were due to the magnitude of the observed effect. Lead size was not an independent predictor of risk of lead failure if lead type was taken into account. Last, not enough data were retrieved to assess the possible modulating effect of single versus dual coil and number of leads (ie, comparison of single‐, dual‐, and triple‐chamber devices; <10 studies provided this information, which prevented us from performing meta–regression analysis).

## Conclusions

All assessed ICD lead families present with failure. The Durata, Endotak Reliance, and Sprint Quattro leads displayed low annual incidence of failure (0.29%–0.45% per year) in the first few years after implant (i.e., in the best case scenario, 2.9% to 4.5% failure can eventually be expected in the first 10 years). There is less experience and data regarding the most recent lead, Durata. This first analysis of studies with short follow‐up duration is still not enough to provide full reassurance regarding the Durata lead family, for which a nonsignificant but numerically higher (small difference of 0.09%–0.16% per year) incidence of lead failure is suggested. We do not know how these low figures will be affected by new data coming from studies with longer follow‐up duration. The Riata and Sprint Fidelis leads were associated with 3‐ and 8‐fold increases in events, respectively, compared with the nonrecalled leads.

Evidence is still scarce regarding mid‐ and long‐term (beyond 5–10 years after implant) performance of ICD leads, and further studies should be conducted to address this problem, ideally, with an agreed international standard to ensure comparable outcome analysis.

## Sources of Funding

Research supported by University College of London Hospitals Biomedicine Research Centre, a Partnership between University College of London and University College of London Hospitals NHS Trust, funded by the National Institute for Health Research (NIHR).

## Disclosures

Providência received training grants from Boston Scientific and Sorin Medical and a Research Grant from Medtronic; Novak received a training grant from Boston Scientific; Lambiase Research grants and speaker fees from Boston Scientific, St Jude, Research Grants from Medtronic and Biotronik, Funding from UCLH Biomedicine NIHR.

## Supporting information


**Figure S1.** Forest plot comparing the incidence of lead failure in the Endotak Reliance and Sprint Quattro lead families.
**Figure S2.** Forest plot comparing the incidence of lead failure in the Sprint Quattro and Durata lead families.
**Figure S3.** Forest plot comparing the incidence of lead failure in the Endotak Reliance and Durata lead families.
**Figure S4.** Forest plot comparing the incidence of lead failure in the Riata and Sprint Fidelis lead families.
**Figure S5.** Forest plot comparing the incidence of lead failure in the Sprint Quattro and Sprint Fidelis lead families.
**Figure S6.** Forest plot comparing the incidence of lead failure in the Durata and Riata lead families.
**Figure S7.** Forest plot comparing the incidence of lead failure in the Endotak Reliance and Riata lead families.
**Figure S8.** Forest plot comparing the incidence of lead failure in the Endotak Reliance and Sprint Fidelis lead families.
**Figure S9.** Forest plot comparing the incidence of lead failure in the Sprint Quattro and Riata lead families.
**Figure S10.** Forest plot comparing the incidence of lead failure in the Durata and Sprint Fidelis lead families.
**Figure S11.** Funnel plots assessing study selection bias in the recalled vs nonrecalled (A) and 7‐vs ≥8‐French (B) sensitivity analyses.
**Figure S12.** Meta–regression plots assessing the association of age, rate of male participants, rate of CRT use, and mean follow‐up duration in the incidence of lead failure in the 8‐ vs 7‐French sensitivity analysis.
**Table S1.** Assessment of the Quality of Included Studies: Newcastle‐Ottawa Scale for Cohort Studies
**Table S2.** Head‐to‐Head Comparison of the 5 Lead Families: Sensitivity Analysis Excluding Studies Featuring Mechanical Failure as Part of the Combined End Point

## References

[1] Mirowski M , Reid PR , Mower MM , Watkins L , Gott VL , Schauble JF , Langer A , Heilman MS , Kolenik SA , Fischell RE , Weisfeldt ML . Termination of malignant ventricular arrhythmias with an implanted automatic defibrillator in human beings. N Engl J Med. 1980;303:322–324. 6991948 10.1056/NEJM198008073030607

[2] Maisel WH , Kramer DB . Implantable cardioverter‐defibrillator lead performance. Circulation. 2008;117:2721–2723. 18506015 10.1161/CIRCULATIONAHA.108.776807

[3] The Antiarrhythmics versus Implantable Defibrillators (AVID) Investigators . A comparison of antiarrhythmic drug therapy with implantable defibrillators in patients resuscitated from near‐fatal ventricular arrhythmias. N Engl J Med. 1997;337:1576–1583. 9411221 10.1056/NEJM199711273372202

[4] Kuck KH , Cappato R , Siebels J , Rüppel R . Randomized comparison of antiarrhythmic drug therapy with implantable defibrillators in patients resuscitated from cardiac arrest: the Cardiac Arrest Study Hamburg (CASH). Circulation. 2000;102:748–754. 10942742 10.1161/01.cir.102.7.748

[5] Connolly SJ , Gent M , Roberts RS , Dorian P , Roy D , Sheldon RS , Mitchell LB , Green MS , Klein GJ , O'Brien B . Canadian implantable defibrillator study (CIDS): a randomized trial of the implantable cardioverter defibrillator against amiodarone. Circulation. 2000;101:1297–1302. 10725290 10.1161/01.cir.101.11.1297

[6] Moss AJ , Hall WJ , Cannom DS , Daubert JP , Higgins SL , Klein H , Levine JH , Saksena S , Waldo AL , Wilber D , Brown MW , Heo M . Improved survival with an implanted defibrillator in patients with coronary disease at high risk for ventricular arrhythmia. Multicenter Automatic Defibrillator Implantation Trial Investigators. N Engl J Med. 1996;335:1933–1940. 8960472 10.1056/NEJM199612263352601

[7] Buxton AE , Lee KL , Fisher JD , Josephson ME , Prystowsky EN , Hafley G . A randomized study of the prevention of sudden death in patients with coronary artery disease. Multicenter Unsustained Tachycardia Trial Investigators. N Engl J Med. 1999;341:1882–1890. 10601507 10.1056/NEJM199912163412503

[8] Moss AJ , Zareba W , Hall WJ , Klein H , Wilber DJ , Cannom DS , Daubert JP , Higgins SL , Brown MW , Andrews ML ; Multicenter Automatic Defibrillator Implantation Trial II Investigators . Prophylactic implantation of a defibrillator in patients with myocardial infarction and reduced ejection fraction. N Engl J Med. 2002;346:877–883. 11907286 10.1056/NEJMoa013474

[9] Bardy GH , Lee KL , Mark DB , Poole JE , Packer DL , Boineau R , Domanski M , Troutman C , Anderson J , Johnson G , McNulty SE , Clapp‐Channing N , Davidson‐Ray LD , Fraulo ES , Fishbein DP , Luceri RM , Ip JH ; Sudden Cardiac Death in Heart Failure Trial (SCD‐HeFT) Investigators . Amiodarone or an implantable cardioverter‐defibrillator for congestive heart failure. N Engl J Med. 2005;352:225–237. 15659722 10.1056/NEJMoa043399

[10] Bristow MR , Saxon LA , Boehmer J , Krueger S , Kass DA , De MT , Carson P , DiCarlo L , DeMets D , White BG , DeVries DW , Feldman AM ; Comparison of Medical Therapy, Pacing, and Defibrillation in Heart Failure (COMPANION) Investigators . Cardiac‐resynchronization therapy with or without an implantable defibrillator in advanced chronic heart failure. N Engl J Med. 2004;350:2140–2150. 15152059 10.1056/NEJMoa032423

[11] Maisel WH . Transvenous implantable cardioverter‐defibrillator leads: the weakest link. Circulation. 2007;115:2461–2463. 17502585 10.1161/CIRCULATIONAHA.107.698597

[12] United States Food and Drug Administration . Safety: Medtronic Sprint Fidelis defibrillator leads. Available at: http://www.fda.gov/safety/medwatch/safetyinformation/safetyalertsforhumanmedicalproducts/ucm152658.htm. Accessed August 8, 2014.

[13] United States Food and Drug Administration . Safety: St. Jude Medical, Riata and Riata ST silicone endocardial defibrillation leads: Class 1 recall ‐ failures with lead insulation. Available at: http://www.fda.gov/Safety/MedWatch/SafetyInformation/SafetyAlertsforHumanMedicalProducts/ucm284390.htm. Accessed August 8, 2014.

[14] United States Food and Drug Administration . Safety: Premature insulation failure in recalled Riata implantable cardioverter defibrillator (ICD) leads manufactured by St. Jude Medical, Inc. Available at: http://www.fda.gov/MedicalDevices/Safety/AlertsandNotices/ucm314930.htm. Accessed August 8, 2014.

[15] United States Food and Drug Administration . Medtronic recalls Sprint Fidelis cardiac leads: Questions and answers for consumers. Available at: http://www.fda.gov/ForConsumers/ConsumerUpdates/ucm103022.htm. Accessed August 8, 2014.

[16] Wilkoff BL , Styperek R , Kloosterman M , Greenberg S , Wong W , Jumrussirikul P . Performance of Optim, Sprint Quattro, and Endotak Reliance Leads. Heart Rhythm. 2013;10:S116–S117.

[17] Rordorf R , Poggio L , Savastano S , Vicentini A , Petracci B , Chieffo E , Klersy C , Landolina M . Failure of implantable cardioverter‐defibrillator leads: a matter of lead size? Heart Rhythm. 2013;10:184–190. 23063430 10.1016/j.hrthm.2012.10.017

[18] Moher D , Liberati A , Tetzlaff J , Altman DG ; PRISMA Group . Preferred reporting items for systematic reviews and meta‐analyses: the PRISMA statement. Ann Intern Med. 2009;151:264–269. 19622511 10.7326/0003-4819-151-4-200908180-00135

[19] Wells G , Shea B , O'Connell D , Peterson J , Welch V , Losos M , Tugwell P . The Newcastle‐Ottawa Scale (NOS) for Assessing the Quality of Nonrandomised Studies in Meta‐analysis. Available at: http://www.ohri.ca/programs/clinical_epidemiology/oxford.htm. Accessed August 8, 2015

[20] Higgins JPT, Green S, eds. Cochrane Handbook for Systematic Reviews of Interventions Version 5.1.0 [updated March 2011]. The Cochrane Collaboration, 2011 Available at: www.cochrane-handbook.org. Accessed August 8, 2014.

[21] Kleemann T , Becker T , Doenges K , Vater M , Senges J , Schneider S , Saggau W , Weisse U , Seidl K . Annual rate of transvenous defibrillation lead defects in implantable cardioverter‐defibrillators over a period of >10 years. Circulation. 2007;115:2474–2480. 17470696 10.1161/CIRCULATIONAHA.106.663807

[22] Eckstein J , Koller MT , Zabel M , Kalusche D , Schaer BA , Osswald S , Sticherling C . Necessity for surgical revision of defibrillator leads implanted long‐term: causes and management. Circulation. 2008;117:2727–2733. 18490526 10.1161/CIRCULATIONAHA.107.740670

[23] Hauser RG , Maron BJ , Marine JE , Lampert R , Kadish AH , Winters SL , Scher DL , Biria M , Kalia A . Safety and efficacy of transvenous high‐voltage implantable cardioverter‐defibrillator leads in high‐risk hypertrophic cardiomyopathy patients. Heart Rhythm. 2008;5:1517–1522. 18984525 10.1016/j.hrthm.2008.08.021

[24] Borleffs CJ , van Erven L , van Bommel RJ , van der Velde ET , van der Wall EE , Bax JJ , Rosendaal FR , Schalij MJ . Risk of failure of transvenous implantable cardioverter‐defibrillator leads. Circ Arrhythm Electrophysiol. 2009;2:411–416. 19808497 10.1161/CIRCEP.108.834093

[25] Hauser RG , Hayes DL . Increasing hazard of Sprint Fidelis implantable cardioverter‐defibrillator lead failure. Heart Rhythm. 2009;6:605–610. 19285920 10.1016/j.hrthm.2009.02.024

[26] Morrison TB , Friedman PA , Kallinen LM , Hodge DO , Crusan D , Kumar K , Hayes DL , Rea RF , Hauser RG . Impact of implanted recalled sprint Fidelis lead on patient mortality. J Am Coll Cardiol. 2011;58:278–283. 21737019 10.1016/j.jacc.2011.03.027

[27] Parkash R , Thibault B , Sterns L , Sapp J , Krahn A , Talajic M , Luce M , Yetisir E , Theoret‐Patrick P , Wells G , Tang A . Sprint Fidelis lead fractures in patients with cardiac resynchronization therapy devices: insight from the Resynchronization/Defibrillation for Ambulatory Heart Failure (RAFT) study. Circulation. 2012;126:2928–2934. 23159551 10.1161/CIRCULATIONAHA.112.132100

[28] Sung RK , Massie BM , Varosy PD , Moore H , Rumsfeld J , Lee BK , Keung E . Long‐term electrical survival analysis of Riata and Riata ST silicone leads: National Veterans Affairs experience. Heart Rhythm. 2012;9:1954–1961. 22871583 10.1016/j.hrthm.2012.08.006

[29] Abdelhadi RH , Saba SF , Ellis CR , Mason PK , Kramer DB , Friedman PA , Gura MT , DiMarco JP , Mugglin AS , Reynolds MR , Bazaz RR , Retel LK , Hayes DL , Hauser RG . Independent multicenter study of Riata and Riata ST implantable cardioverter‐defibrillator leads. Heart Rhythm. 2013;10:361–365. 23128017 10.1016/j.hrthm.2012.10.045

[30] Ellenbogen KA , Gunderson BD , Stromberg KD , Swerdlow CD . Performance of Lead Integrity Alert to assist in the clinical diagnosis of implantable cardioverter defibrillator lead failures: analysis of different implantable cardioverter defibrillator leads. Circ Arrhythm Electrophysiol. 2013;6:1169–1177. 24099976 10.1161/CIRCEP.113.000744

[31] Fazal IA , Shepherd EJ , Tynan M , Plummer CJ , McComb JM . Comparison of Sprint Fidelis and Riata defibrillator lead failure rates. Int J Cardiol. 2013;168:848–852. 23138013 10.1016/j.ijcard.2012.10.015

[32] Verlato R , Facchin D , Catanzariti D , Molon G , Zanotto G , Morani G , Brieda M , Zanon F , Delise P , Leoni L , Comisso J , Campo C . Clinical outcomes in patients with implantable cardioverter defibrillators and Sprint Fidelis leads. Heart. 2013;99:799–804. 23434626 10.1136/heartjnl-2012-303259

[33] Liu J , Brumberg G , Rattan R , Patel D , Adelstein E , Jain S , Saba S . Longitudinal follow‐up of implantable cardioverter defibrillator leads. Am J Cardiol. 2014;113:103–106. 24176060 10.1016/j.amjcard.2013.08.046

[34] Vollmann D , Woronowicz S , Kmiec L , Jung K , Zenker D , Seegers J , Sossalla S , Dorenkamp M , Sohns C , Lüthje L , Hasenfuss G , Zabel M . Passive‐fixation lead failure rates and long‐term patient mortality in subjects implanted with Sprint Fidelis electrodes. Europace. 2014;16:258–264. 23813451 10.1093/europace/eut185

[35] Yanagisawa S , Inden Y , Shimano M , Yoshida N , Ichiyanagi H , Fujita M , Ohguchi S , Ishikawa S , Kato H , Okumura S , Miyoshi A , Nagao T , Yamamoto T , Hirai M , Murohara T . Clinical outcome of implantable cardioverter defibrillators with recalled and non‐recalled leads in Japanese patients. Increased failure rate of the Sprint Fidelis lead. Circ J. 2014;78:353–359. 24270680 10.1253/circj.cj-13-1040

[36] Kramer DB , Hatfield LA , McGriff D , Ellis CR , Gura MT , Samuel M , Retel LK , Hauser RG . Transvenous Implantable Cardioverter‐defibrillator Lead Reliability: implications for Post‐Market Surveillance. J Am Heart Assoc. 2015;4:e001672 doi: 10.1161/JAHA.114.001672. 26025935 10.1161/JAHA.114.001672 PMC4599526

[37] Hauser RG , Kallinen LM , Almquist AK , Gornick CC , Katsiyiannis WT . Early failure of a small‐diameter high‐voltage implantable cardioverter‐defibrillator lead. Heart Rhythm. 2007;4:892–896. 17599673 10.1016/j.hrthm.2007.03.041

[38] Medtronic, Inc . Medtronic Technical Concept Paper: Insights on Sprint Design Enhancements. June 2004 Medtronic Product Performance Report. Available at: http://wwwp.medtronic.com/productperformance/. Accessed September 21, 2014.

[39] Endotak Reliance Product Information . Boston Scientific lead information. Available at: http://www.bostonscientific.com/en-US/products/leads.html. Accessed April 10, 2015.

[40] Boston Scientific CRM Product Performance Report 2014—Q4 Edition. Available at: http://www.bostonscientific.com/content/dam/bostonscientific/quality/ppr/2014/Q4/Product%20Performance%20Report%20Q4%202014%20Rev%20E.pdf. Accessed April 11, 2015.

[41] Hauser RG , McGriff D , Retel LK . Riata implantable cardioverter‐defibrillator lead failure: analysis of explanted leads with a unique insulation defect. Heart Rhythm. 2012;9:742–749. 22209723 10.1016/j.hrthm.2011.12.019

[42] St. Jude Medical ICD lead design and long‐term performance. Available at: http://professional-intl.sjm.com/products/crm/leads/defibrillation-leads/durata-defibrillation#overview. Accessed September 21, 2014.

[43] St. Jude Medical Product performance report. 1st Edition, May 2014 Available at: http://sjm.com/~/media/pro/resources/product-performance/reports/2015_1sted_050715.ashx. Accessed September 21, 2014.

[44] Zhao X , Thomann S , Alfalasi O , Massin F , Cransac F , Cung T , Davy JM , Pasquie JL . Riata silicone defibrillation leads failure: increase in prevalence after 5 years of follow‐up. Heart Rhythm. 2014;11(suppl):S504–S505.

[45] Cairns JA , Pogue J , Connolly SJ , Epstein AE , Healey JS , Buller CE , Themeles E . Optim ICD lead failures: long‐term rates from independent analysis of 11,016 leads in 3 prospective registries. Heart Rhythm. 2014;11(suppl):S432–S433.

[46] Faulknier BA , Traub DM , Aktas MK , Aguila A , Rosero S , Daubert JP , Hall B , Shah A , Taylor S , McNitt S , Moss AJ , Zareba W , Huang DT . Time‐dependent risk of Fidelis lead failure. Am J Cardiol. 2010;105:95–99. 20102898 10.1016/j.amjcard.2009.08.655

[47] Theuns DA , Elvan A , de Voogt W , de Cock CC , van Erven L , Meine M . Prevalence and presentation of externalized conductors and electrical abnormalities in Riata defibrillator leads after fluoroscopic screening: report from the Netherlands Heart Rhythm Association Device Advisory Committee. Circ Arrhythm Electrophysiol. 2012;5:1059–1063. 23091049 10.1161/CIRCEP.112.975755

[48] Hauser RG . Here we go again–failure of postmarketing device surveillance. N Engl J Med. 2012;366:873–875. 22332951 10.1056/NEJMp1114695

[49] Hauser RG . Please leave the reliable ICD leads alone. Heart Rhythm. 2013;10:562–563. 23274369 10.1016/j.hrthm.2012.12.024

[50] United States Food and Drug Administration . Unique Device Identification – UDI. Available at http://www.fda.gov/MedicalDevices/DeviceRegulationandGuidance/UniqueDeviceIdentification/default.htm. Accessed April 11, 2015.

[51] Lambiase PD , Barr C , Theuns DA , Knops R , Neuzil P , Johansen JB , Hood M , Pedersen S , Kääb S , Murgatroyd F , Reeve HL , Carter N , Boersma L ; EFFORTLESS Investigators . Worldwide experience with a totally subcutaneous implantable defibrillator: early results from the EFFORTLESS S‐ICD Registry. Eur Heart J. 2014;35:1657–1665. 24670710 10.1093/eurheartj/ehu112 PMC4076663

