# Supplementary material for: Transvenous Implantable Cardioverter‐Defibrillator (ICD) Lead Performance: A Meta‐Analysis of Observational Studies
Source: J Am Heart Assoc. 2015 Oct 30;4(11):e002418. doi: 10.1161/JAHA.115.002418 (PMC4845221; doi:10.1161/JAHA.115.002418)
Supplement: Supplementary file 1 — Figure S1. Forest plot comparing the incidence of lead failure in the Endotak Reliance and Sprint Quattro lead families. Figure S2. Forest plot comparing the incidence of lead failure in the Sprint Quattro and Durata lead families. Figure S3. Forest plot comparing the incidence of lead failure in the Endotak Reliance and Durata lead families. Figure S4. Forest plot comparing the incidence of lead failure in the Riata and Sprint Fidelis lead families. Figure S5. Forest plot comparing the incidence of lead failure in the Sprint Quattro and Sprint Fidelis lead families. Figure S6. Forest plot comparing the incidence of lead failure in the Durata and Riata lead families. Figure S7. Forest plot comparing the incidence of lead failure in the Endotak Reliance and Riata lead families. Figure S8. Forest plot comparing the incidence of lead failure in the Endotak Reliance and Sprint Fidelis lead families. Figure S9. Forest plot comparing the incidence of lead failure in the Sprint Quattro and Riata lead families. Figure S10. Forest plot comparing the incidence of lead failure in the Durata and Sprint Fidelis lead families. Figure S11. Funnel plots assessing study selection bias in the recalled vs nonrecalled (A) and 7‐vs ≥8‐French (B) sensitivity analyses. Figure S12. Meta–regression plots assessing the association of age, rate of male participants, rate of CRT use, and mean follow‐up duration in the incidence of lead failure in the 8‐ vs 7‐French sensitivity analysis. Table S1. Assessment of the Quality of Included Studies: Newcastle‐Ottawa Scale for Cohort Studies Table S2. Head‐to‐Head Comparison of the 5 Lead Families: Sensitivity Analysis Excluding Studies Featuring Mechanical Failure as Part of the Combined End Point [file JAH3-4-e002418-s001.pdf]

Supplementary Material

Figure S-1 – Forest plot comparing the incidence of lead failure in the Endotak Reliance vs. Sprint Quattro lead families.

	Endotak			Quattro				Mean Difference	
Study or Subgroup	Mean	SD	Total	Mean	SD	Total	Weight	IV, Random, 95% CI	Year
Kleeman 2007	0	9.42	1	0	7.25	139	0.0%	0.00 [-18.50, 18.50]	2007
Hauser 2008	0.81	6.45	89	0	4.2	70	1.2%	0.81 [-0.85, 2.47]	2008
Eckstein 2008	0.1	1.7	159	0.46	3.06	103	6.8%	-0.36 [-1.01, 0.29]	2008
Hauser 2009	0.56	5.59	607	0.55	6.1	1273	8.7%	0.01 [-0.55, 0.57]	2009
Borleffs 2009	0.77	6.08	911	1.56	6.32	322	4.8%	-0.79 [-1.59, 0.01]	2010
Sung 2012	0.16	2.38	2401	0.15	2.39	6091	36.1%	0.01 [-0.10, 0.12]	2012
Rordorf 2013	1.06	6.01	204	0	2.62	130	3.5%	1.06 [0.12, 2.00]	2013
Liu 2014	0.26	2.39	2190	0.31	3.1	1020	26.6%	-0.05 [-0.26, 0.16]	2014
Kramer 2015	0.43	3.67	389	0.15	5.55	1818	12.2%	0.28 [-0.17, 0.73]	2015
**Total (95% CI)**			**6951**			**10966**	**100.0%**	**0.01 [-0.17, 0.19]**	

Heterogeneity: $Tau^2 = 0.02$; $Chi^2 = 12.53$, df = 8 (P = 0.13); $I^2 = 36\%$

Test for overall effect: Z = 0.11 (P = 0.91)

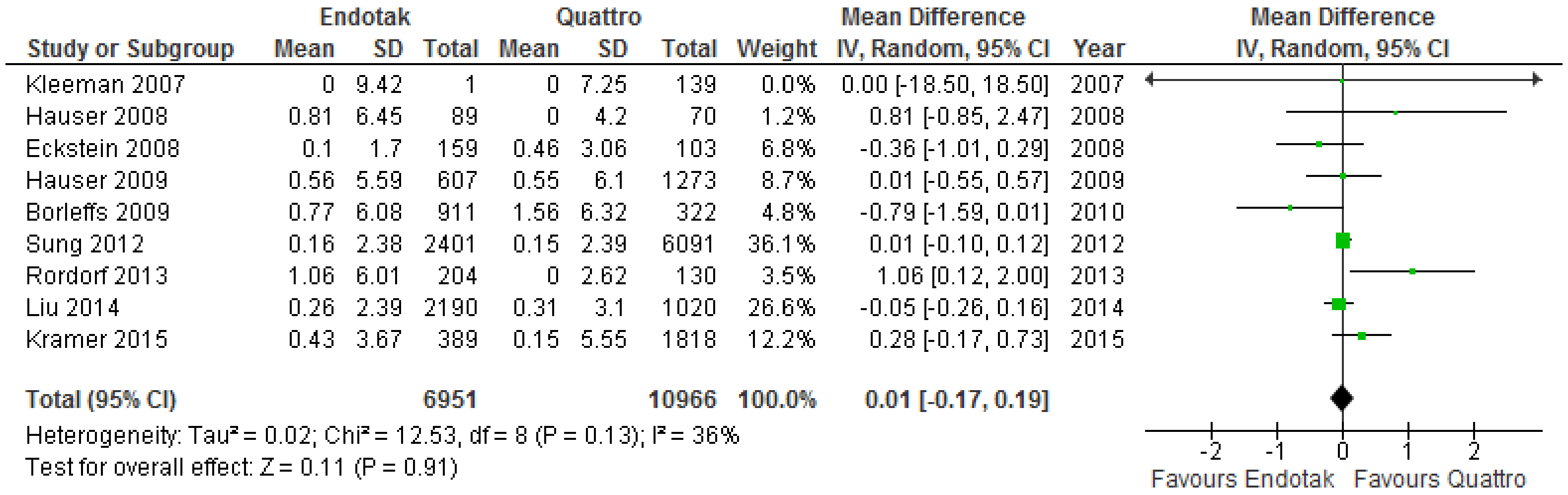

Figure S-2 – Forest plot comparing the incidence of lead failure in the Sprint Quattro vs. Durata lead families.

	Quattro			Durata				Mean Difference	
Study or Subgroup	Mean	SD	Total	Mean	SD	Total	Weight	IV, Random, 95% CI	Year
Rordorf 2013	0	2.62	130	0	5.63	86	12.3%	0.00 [-1.27, 1.27]	2013
Liu 2014	0.31	3.1	1020	0.95	6.53	828	39.8%	-0.64 [-1.12, -0.16]	2014
Kramer 2015	0.15	5.55	1818	0.15	2.74	445	47.9%	0.00 [-0.36, 0.36]	2015
**Total (95% CI)**			**2968**			**1359**	**100.0%**	**-0.25 [-0.75, 0.24]**	

Heterogeneity: Tau² = 0.10; Chi² = 4.44, df = 2 (P = 0.11); I² = 55%
Test for overall effect: Z = 1.01 (P = 0.31)

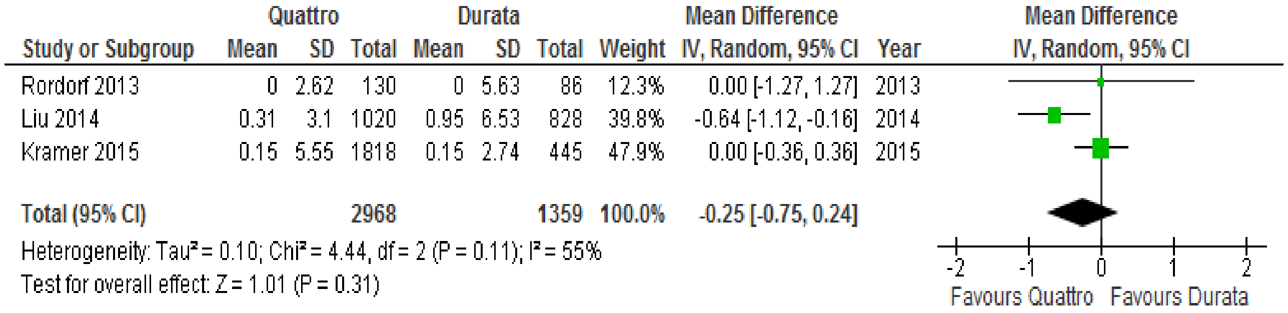

Figure S-3 – Forest plot comparing the incidence of lead failure in the Endotak Reliance vs. Durata lead families.

	Endotak			Durata				Mean Difference	
Study or Subgroup	Mean	SD	Total	Mean	SD	Total	Weight	IV, Random, 95% CI	Year
Rordorf 2013	1.06	6.01	204	0	5.63	86	12.1%	1.06 [-0.39, 2.51]	2013
Ellenbogen 2013	0.73	9.1	3654	0.3	6.43	4179	30.5%	0.43 [0.08, 0.78]	2013
Liu 2014	0.26	2.39	2190	0.95	6.53	828	28.6%	-0.69 [-1.15, -0.23]	2014
Kramer 2015	0.43	3.67	389	0.15	2.74	445	28.8%	0.28 [-0.16, 0.72]	2015
**Total (95% CI)**			**6437**			**5538**	**100.0%**	**0.14 [-0.48, 0.77]**	

Heterogeneity: Tau² = 0.30; Chi² = 17.19, df = 3 (P = 0.0006); I² = 83%
Test for overall effect: Z = 0.44 (P = 0.66)

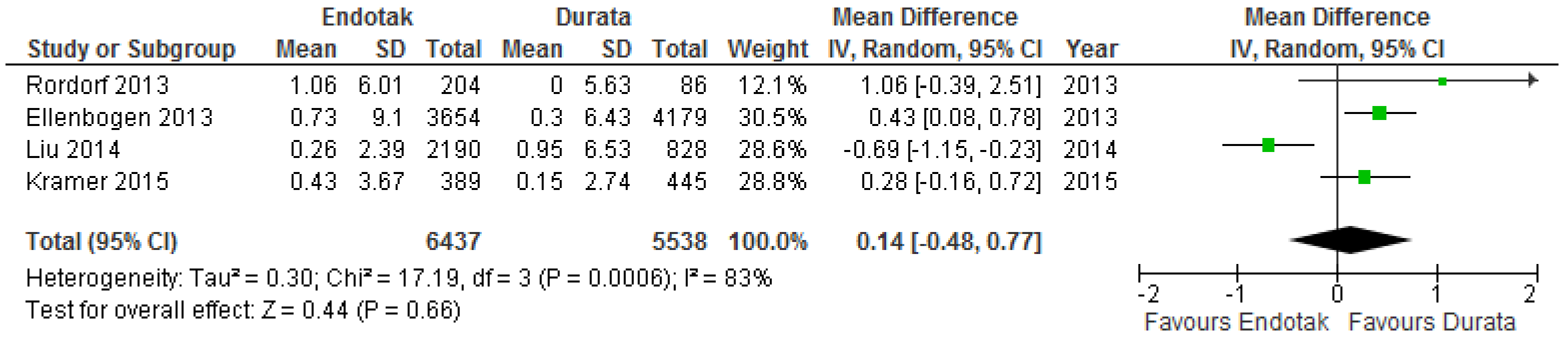

Figure S-4 – Forest plot comparing the incidence of lead failure in the Riata vs. Sprint Fidelis lead families.

	Riata			Fidelis				Mean Difference	
Study or Subgroup	Mean	SD	Total	Mean	SD	Total	Weight	IV, Random, 95% CI	Year
Kleeman 2007	7.78	29.69	199	0	23.83	1	0.0%	7.78 [-39.11, 54.67]	2007
Hauser 2008	2.45	18.44	35	3.92	17.19	69	1.2%	-1.47 [-8.80, 5.86]	2008
Hauser 2009	0.47	6.12	90	3.74	12.7	848	12.3%	-3.27 [-4.80, -1.74]	2009
Borleffs 2009	0.79	5.9	183	2.74	13.41	376	11.8%	-1.95 [-3.55, -0.35]	2010
Sung 2012	0.67	3.73	1403	2.08	7.63	5073	20.1%	-1.41 [-1.70, -1.12]	2012
Rordorf 2013	2.54	8.67	182	4.8	10.83	190	9.6%	-2.26 [-4.25, -0.27]	2013
Fazal 2013	2.68	7.8	106	2.61	7.78	113	9.2%	0.07 [-1.99, 2.13]	2013
Ellenbogen 2013	1.2	11.7	1944	0.86	6.94	1556	18.5%	0.34 [-0.28, 0.96]	2013
Yanagisawa 2014	2.55	9.14	8	3.89	9.04	75	1.5%	-1.34 [-8.00, 5.32]	2014
Liu 2014	1.89	7.73	627	2.79	10.12	623	15.9%	-0.90 [-1.90, 0.10]	2014
**Total (95% CI)**			**4777**			**8924**	**100.0%**	**-1.24 [-2.08, -0.40]**	

Heterogeneity: $Tau^2 = 0.90$; $Chi^2 = 36.34$, $df = 9$ ($P < 0.0001$); $I^2 = 75\%$
Test for overall effect: $Z = 2.89$ ($P = 0.004$)

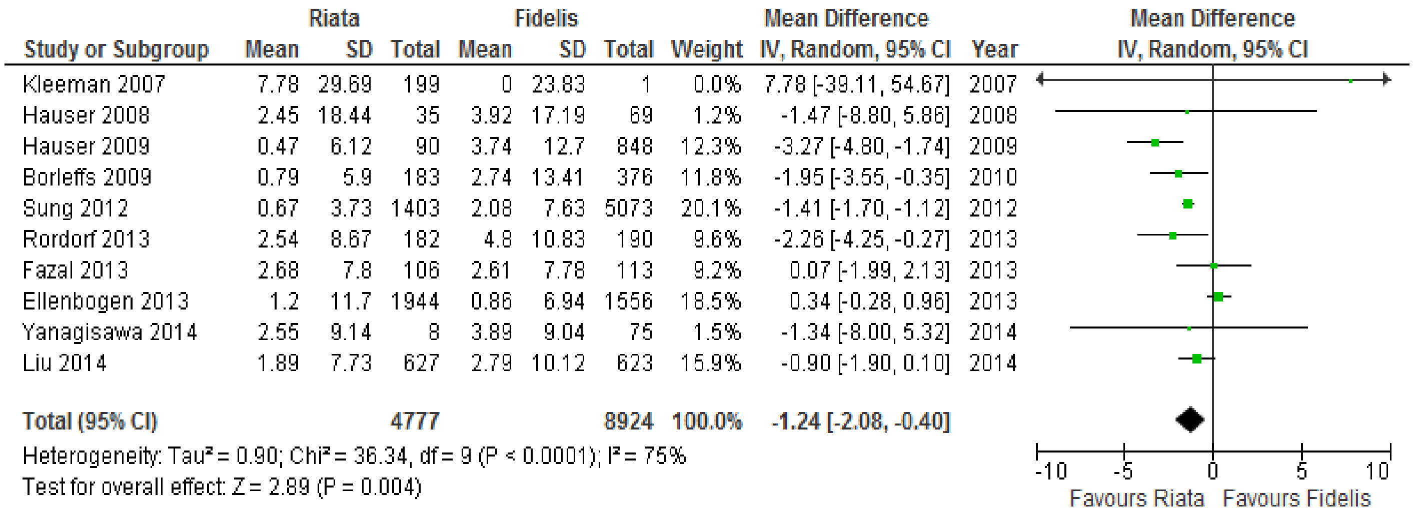

Figure S-5 – Forest plot comparing the incidence of lead failure in the Sprint Quattro vs. Sprint Fidelis lead families.

	Quattro			Fidelis				Mean Difference	
Study or Subgroup	Mean	SD	Total	Mean	SD	Total	Weight	IV, Random, 95% CI	Year
Kleeman 2007	0	7.25	139	0	23.83	1	0.0%	0.00 [-46.72, 46.72]	2007
Hauser 2008	0	4.2	70	3.92	17.19	69	0.9%	-3.92 [-8.09, 0.25]	2008
Hauser 2009	0.55	6.1	1273	3.74	12.7	848	9.6%	-3.19 [-4.11, -2.27]	2009
Borleffs 2009	1.56	6.32	322	2.74	13.41	376	5.2%	-1.18 [-2.70, 0.34]	2010
Morrison 2011	0.42	3.62	1641	2.88	9.99	1030	12.8%	-2.46 [-3.09, -1.83]	2011
Sung 2012	0.15	2.39	6091	2.08	7.63	5073	17.5%	-1.93 [-2.15, -1.71]	2012
Parkash 2012	0.06	1.67	969	1.69	7.22	818	14.4%	-1.63 [-2.14, -1.12]	2012
Rordorf 2013	0	2.62	130	4.8	10.83	190	4.8%	-4.80 [-6.40, -3.20]	2013
Verlato 2013	0.22	4.14	468	1.88	8.22	508	10.8%	-1.66 [-2.47, -0.85]	2013
Vollmann 2014	0.36	2.97	276	2.37	8.56	429	10.0%	-2.01 [-2.89, -1.13]	2014
Yanagisawa 2014	0	2.92	131	3.89	9.04	75	3.2%	-3.89 [-6.00, -1.78]	2014
Liu 2014	0.31	3.1	1020	2.79	10.12	623	10.7%	-2.48 [-3.30, -1.66]	2014
**Total (95% CI)**			**12530**			**10040**	**100.0%**	**-2.29 [-2.71, -1.88]**	

Heterogeneity: $Tau^2 = 0.24$; $Chi^2 = 29.52$, $df = 11$ ($P = 0.002$); $I^2 = 63\%$
Test for overall effect: $Z = 10.92$ ($P < 0.00001$)

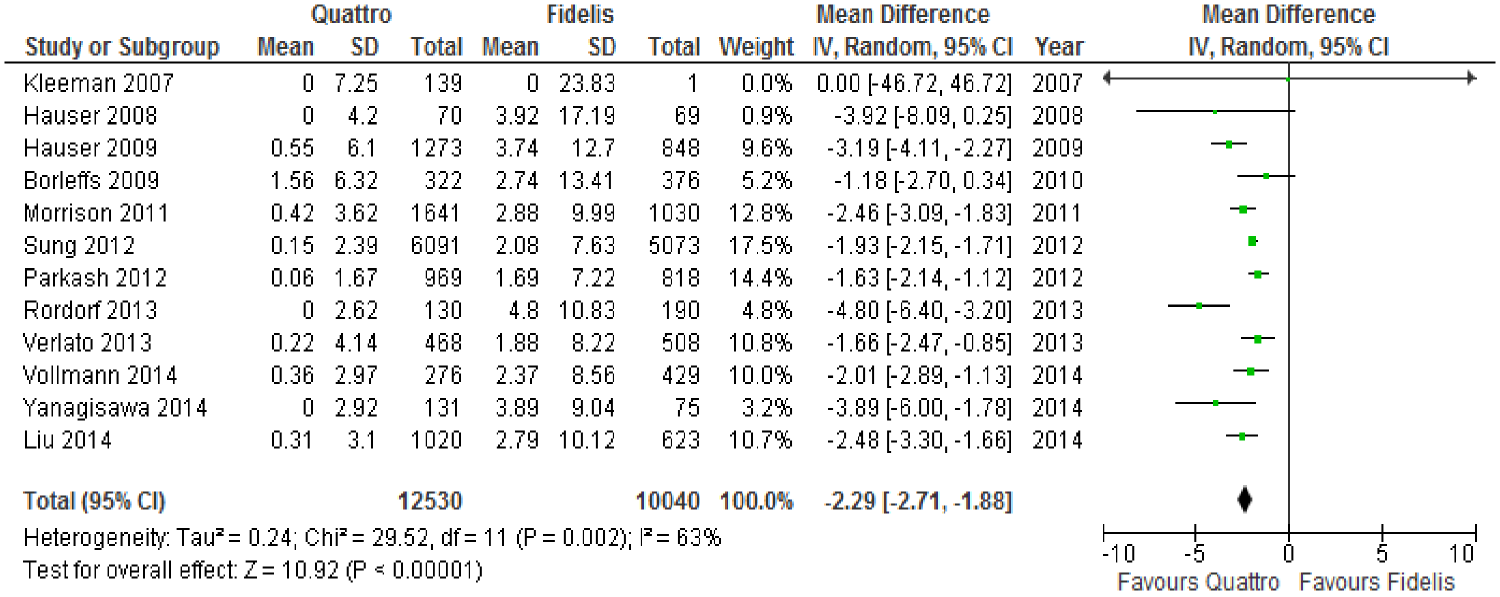

Figure S-6 – Forest plot comparing the incidence of lead failure in the Durata vs. Riata lead families.

Study or Subgroup	Durata Mean	Durata SD	Durata Total	Riata Mean	Riata SD	Riata Total	Weight	Mean Difference IV, Random, 95% CI	Year
Ellenbogen 2013	0.3	6.43	4179	1.2	11.7	1944	43.1%	-0.90 [-1.46, -0.34]	2013
Rordorf 2013	0	5.63	86	2.54	4.09	182	20.6%	-2.54 [-3.87, -1.21]	2013
Liu 2014	0.95	6.53	828	1.89	7.73	627	36.2%	-0.94 [-1.69, -0.19]	2014
**Total (95% CI)**			**5093**			**2753**	**100.0%**	**-1.25 [-2.01, -0.49]**	

Heterogeneity: Tau² = 0.27; Chi² = 5.17, df = 2 (P = 0.08); I² = 61%
Test for overall effect: Z = 3.23 (P = 0.001)

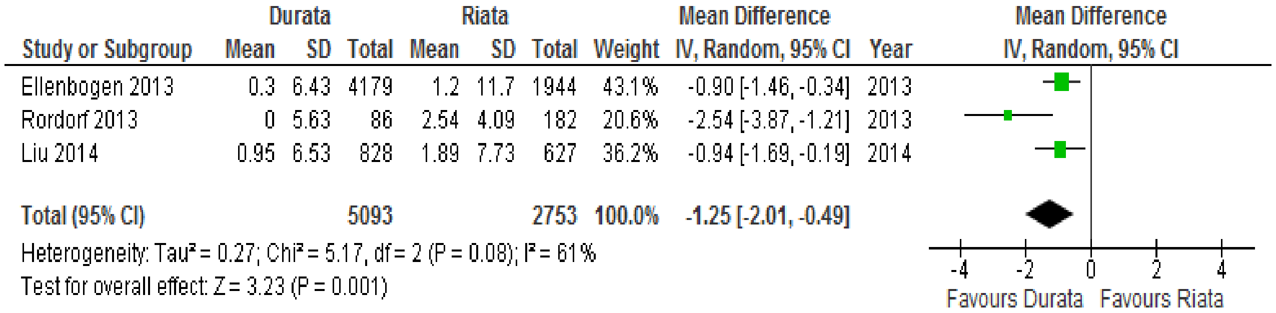

Figure S-7 – Forest plot comparing the incidence of lead failure in the Endotak Reliance vs. Riata lead families.

Study or Subgroup	Endotak Mean	Endotak SD	Endotak Total	Riata Mean	Riata SD	Riata Total	Weight	Mean Difference IV, Random, 95% CI	Year
Kleeman 2007	0	9.42	1	7.78	29.69	199	0.1%	-7.78 [-26.70, 11.14]	2007
Hauser 2008	0.81	6.45	89	2.45	1,844	35	0.0%	-1.64 [-612.55, 609.27]	2008
Eckstein 2008	0.1	1.7	159	0	2.33	42	14.7%	0.10 [-0.65, 0.85]	2008
Hauser 2009	0.56	5.59	607	0.47	6.12	90	7.6%	0.09 [-1.25, 1.43]	2009
Borleffs 2009	0.77	6.08	911	0.79	5.9	183	11.8%	-0.02 [-0.96, 0.92]	2010
Sung 2012	0.16	2.38	2401	0.67	3.73	1403	24.6%	-0.51 [-0.73, -0.29]	2012
Ellenbogen 2013	0.73	9.1	3654	1.2	11.7	1944	17.6%	-0.47 [-1.07, 0.13]	2013
Rordorf 2013	1.06	6.01	204	2.54	8.67	182	6.4%	-1.48 [-2.99, 0.03]	2013
Liu 2014	0.26	2.39	2190	1.89	7.73	627	17.3%	-1.63 [-2.24, -1.02]	2014
**Total (95% CI)**			**10216**			**4705**	**100.0%**	**-0.57 [-1.01, -0.13]**	

Heterogeneity: Tau² = 0.19; Chi² = 19.10, df = 8 (P = 0.01); I² = 58%
Test for overall effect: Z = 2.55 (P = 0.01)

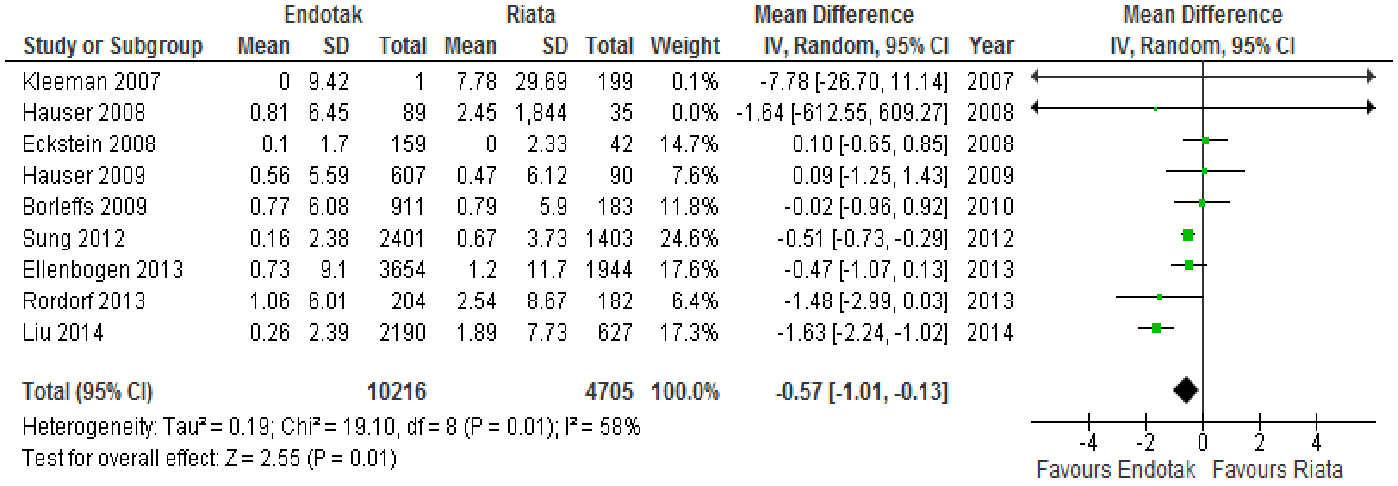

Figure S-8 – Forest plot comparing the incidence of lead failure in the Endotak Reliance vs. Sprint Fidelis lead families.

	Endotak			Fidelis				Mean Difference	
Study or Subgroup	Mean	SD	Total	Mean	SD	Total	Weight	IV, Random, 95% CI	Year
Kleeman 2007	0	9.42	1	0	23.83	1	0.0%	0.00 [-50.22, 50.22]	2007
Hauser 2008	0.81	6.45	89	3.92	17.19	69	3.2%	-3.11 [-7.38, 1.16]	2008
Hauser 2009	0.56	5.59	607	3.74	12.7	848	16.1%	-3.18 [-4.14, -2.22]	2009
Borleffs 2009	0.77	6.08	911	2.74	13.41	376	12.9%	-1.97 [-3.38, -0.56]	2010
Sung 2012	0.16	2.38	2401	2.08	7.63	5073	20.1%	-1.92 [-2.15, -1.69]	2012
Ellenbogen 2013	0.32	6.2	5114	0.86	6.49	1556	19.7%	-0.54 [-0.90, -0.18]	2013
Rordorf 2013	1.06	6.01	204	4.8	10.83	190	10.8%	-3.74 [-5.49, -1.99]	2013
Liu 2014	0.26	2.39	2190	2.79	10.12	623	17.2%	-2.53 [-3.33, -1.73]	2014
**Total (95% CI)**			**11517**			**8736**	**100.0%**	**-2.20 [-3.03, -1.36]**	

Heterogeneity: Tau² = 0.88; Chi² = 61.31, df = 7 (P < 0.00001); I² = 89%

Test for overall effect: Z = 5.16 (P < 0.00001)

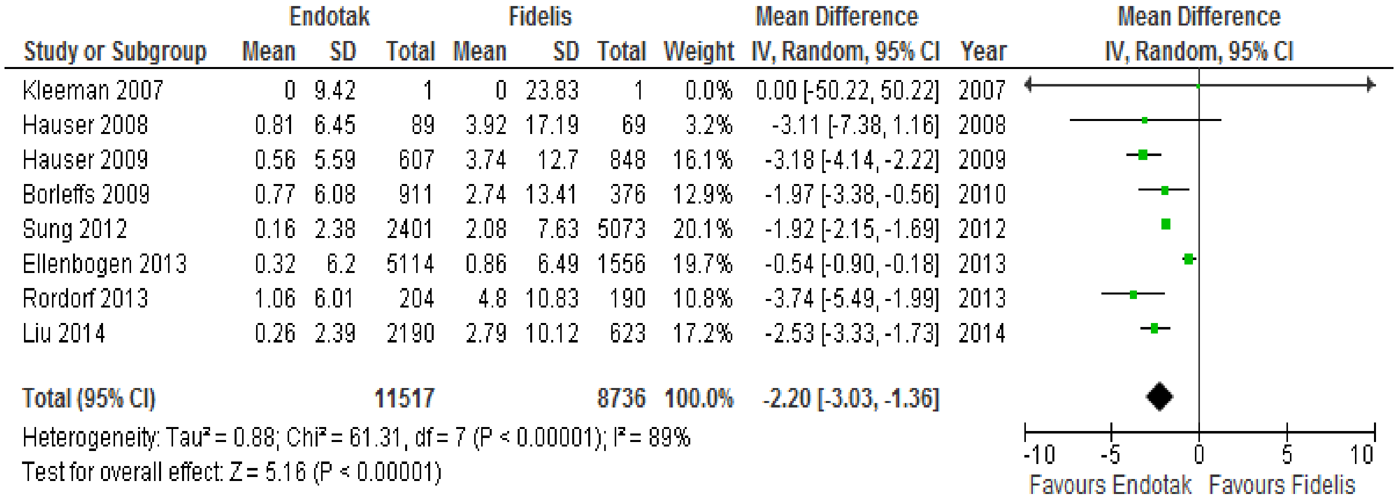

Figure S-9 – Forest plot comparing the incidence of lead failure in the Sprint Quattro vs. Riata lead families.

Study or Subgroup	Quattro Mean	Quattro SD	Quattro Total	Riata Mean	Riata SD	Riata Total	Weight	Mean Difference IV, Random, 95% CI	Year
Kleeman 2007	0	7.25	139	7.78	29.69	199	1.6%	-7.78 [-12.08, -3.48]	2007
Eckstein 2008	0.46	3.06	103	0	2.33	42	13.0%	0.46 [-0.46, 1.38]	2008
Hauser 2008	0	4.2	70	2.45	18.44	35	0.8%	-2.45 [-8.64, 3.74]	2008
Hauser 2009	0.55	6.1	1273	0.47	6.12	90	9.7%	0.08 [-1.23, 1.39]	2009
Borleffs 2009	1.56	6.32	322	0.79	5.9	183	11.4%	0.77 [-0.33, 1.87]	2010
Sung 2012	0.15	2.39	6091	0.67	3.73	1403	19.3%	-0.52 [-0.72, -0.32]	2012
Abdelhadi 2013	0.43	3.75	1668	1.12	5.37	1081	18.2%	-0.69 [-1.06, -0.32]	2013
Rordorf 2013	0	2.62	130	2.54	8.67	182	9.4%	-2.54 [-3.88, -1.20]	2013
Liu 2014	0.31	3.1	1020	1.89	7.73	627	15.9%	-1.58 [-2.21, -0.95]	2014
Yanagisawa 2014	0	2.92	131	2.55	9.14	8	0.8%	-2.55 [-8.90, 3.80]	2014
**Total (95% CI)**			**10947**			**3850**	**100.0%**	**-0.73 [-1.29, -0.16]**	

Heterogeneity: $Tau^2 = 0.43$; $Chi^2 = 41.61$, $df = 9$ ($P < 0.00001$); $I^2 = 78\%$
Test for overall effect: $Z = 2.50$ ($P = 0.01$)

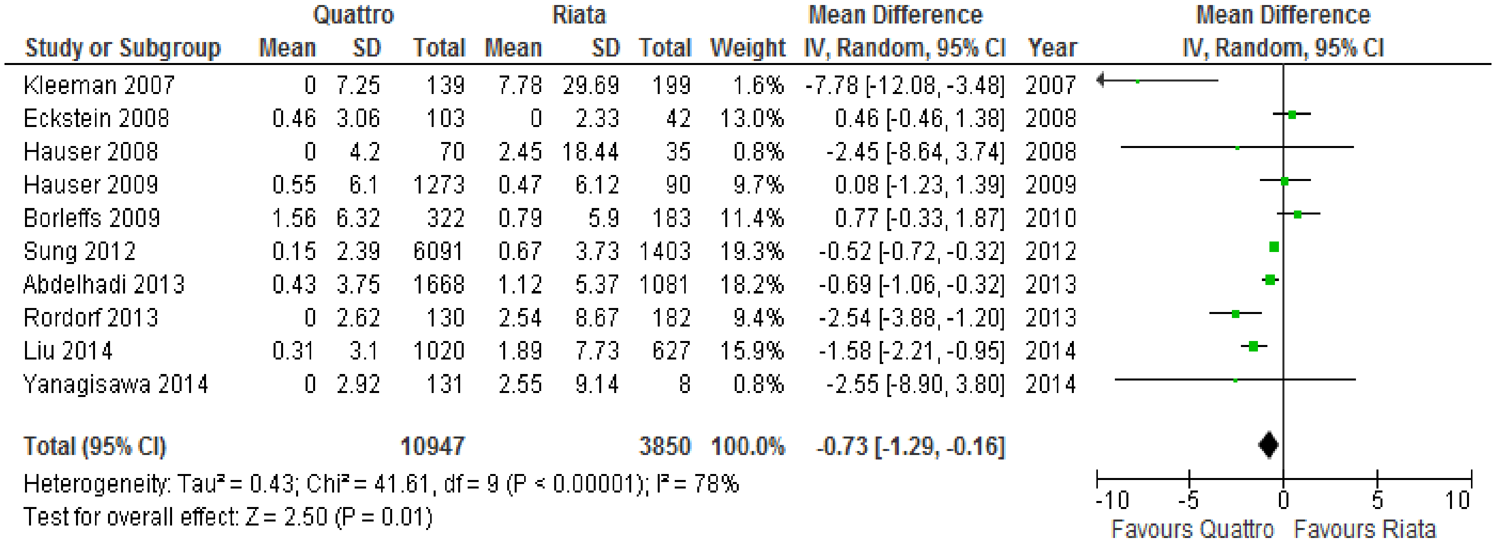

Figure S-10 – Forest plot comparing the incidence of lead failure in the Durata vs. Sprint Fidelis lead families.

	Durata			Fidelis				Mean Difference	
Study or Subgroup	Mean	SD	Total	Mean	SD	Total	Weight	IV, Random, 95% CI	Year
Ellenbogen 2013	0.3	643	4179	0.86	6.94	1556	1.8%	-0.56 [-20.06, 18.94]	2013
Rordorf 2013	0	5.63	86	4.8	10.83	190	44.1%	-4.80 [-6.75, -2.85]	2013
Liu 2014	0.95	6.53	828	2.79	10.12	623	54.1%	-1.84 [-2.75, -0.93]	2014
**Total (95% CI)**			**5093**			**2369**	**100.0%**	**-3.12 [-5.77, -0.47]**	

Heterogeneity: $Tau^2 = 3.17$; $Chi^2 = 7.32$, df = 2 (P = 0.03); $I^2 = 73\%$
Test for overall effect: Z = 2.31 (P = 0.02)

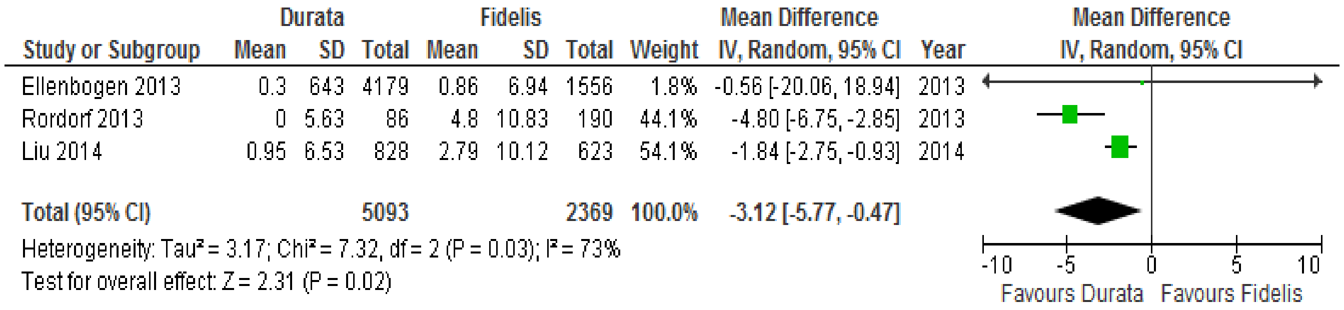

Figure S-11 – Funnel plots assessing study selection bias in the recalled vs non-recalled (Panel A) and 7-French vs ≥ 8-French (Panel B) sensitivity analyses.

A.

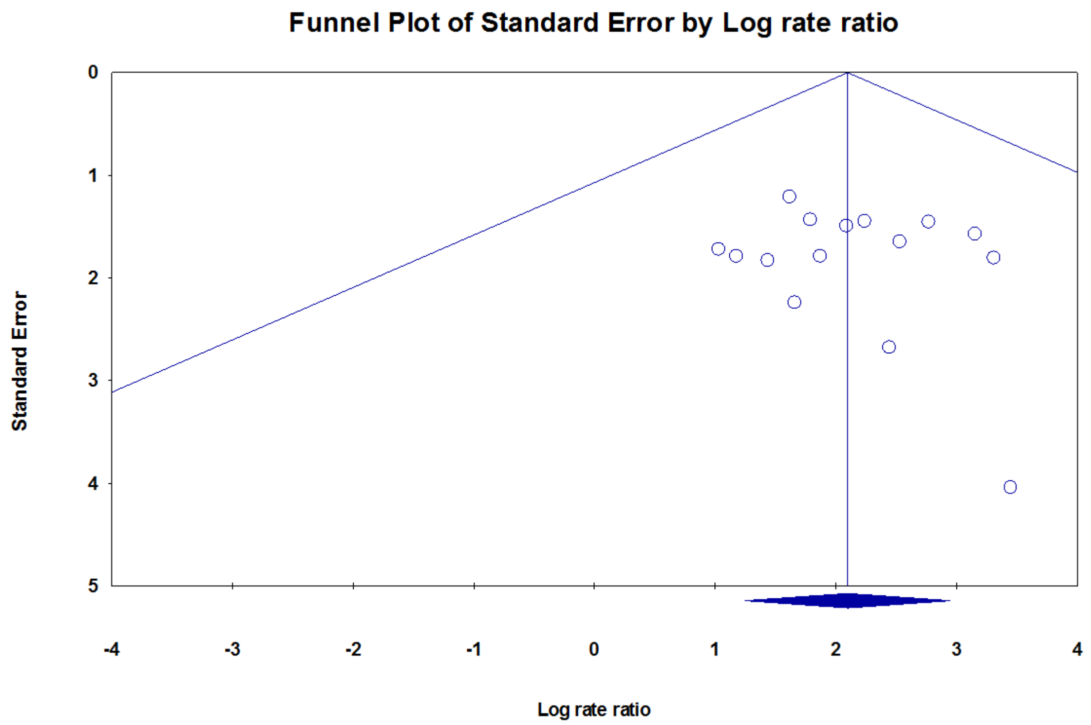

B.

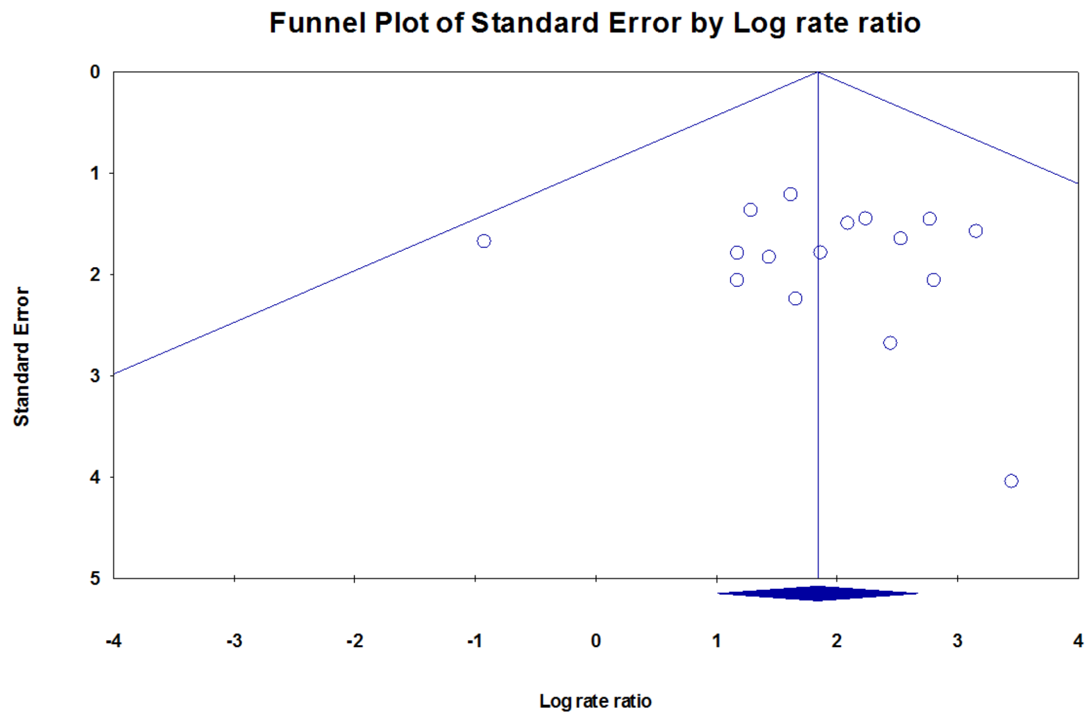

Figure S-12 – Meta-regression plots assessing the association of age, rate of male participants, rate of CRT use and mean follow-up duration in the incidence of lead failure in the 8-French vs. 7-French sensitivity analysis.

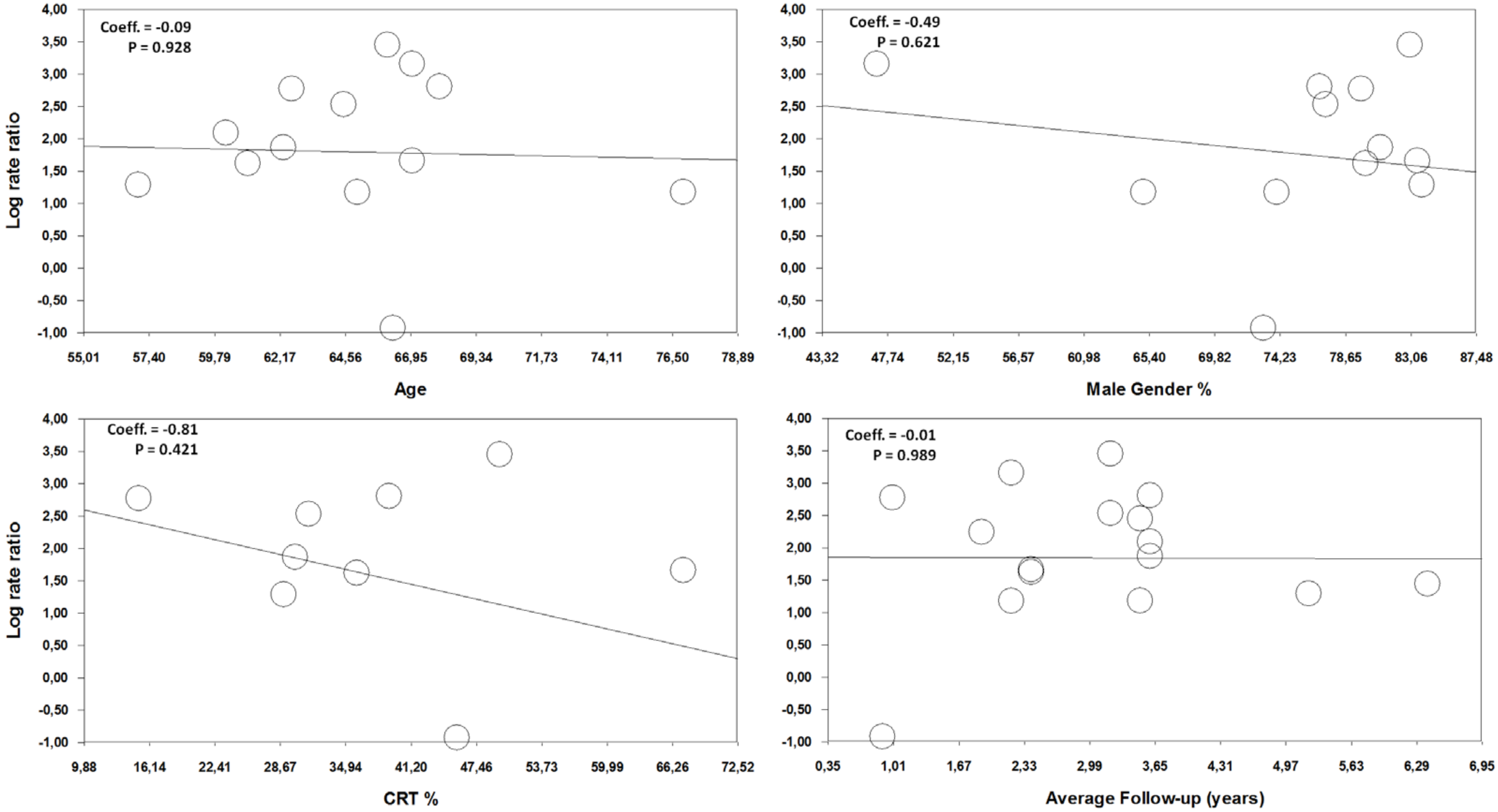

Table S-1 – Assessment of the quality of included studies: Newcastle-Ottawa Scale for Cohort studies.

Newcastle-Ottawa Assessment Scale for Cohort Studies

Article	Selection				Comparability	Outcome			Total of Stars	Comment
	1	2	3	4	1	1	2	3		
Kleeman et al 2007 [1]	+	+	+	+	NA	+	-	+	6	No comparison of baseline variables among different lead families. Mean follow-up < 3 years. % of lost to follow-up is mentioned (<5%) and unlikely to introduce bias.
Eckstein et al 2008 [2]	+	+	+	+	NA	+	+	NA	6	No comparison of baseline variables among different lead families. % of lost to follow-up is not mentioned.
Hauser et al 2008 [3]	+	+	+	+	NA	+	+	-	6	No comparison of baseline variables among different lead families. % of lost to follow-up is mentioned (>5%).
Borleffs et al 2009 [4]	+	+	+	+	NA	+	-	+	6	No comparison of baseline variables among different lead families. Mean follow-up < 3 years. % of lost to follow-up is mentioned (<5%) and unlikely to introduce bias.
Hauser et al 2009 [5]	+	+	+	+	NA	+	-	-	5	No comparison of baseline variables among different lead families. Mean follow-up < 3 years. % of lost to follow-up is mentioned (>5%).
Morrison et al 2011 [6]	+	+	+	+	+	+	+	NA	7	Groups are comparable. % of lost to follow-up is not mentioned.
Parkash et al 2012 [7]	+	+	+	+	+	+	+	NA	7	Groups are comparable. % of lost to follow-up not available.
Sung et al 2012 [8]	+	+	+	+	NA	+	+	NA	6	No comparison of baseline variables among different lead families. % of lost to follow-up is mentioned (>5%).
Abdelhadi et al 2013 [9]	+	+	+	+	-	+	+	NA	6	Baseline differences among the two different lead families. % of lost to follow-up is not mentioned.
Ellenbogen et al 2013 [10]	+	+	+	+	NA	+	-	NA	5	No comparison of baseline variables among different lead families. Mean follow-up < 3 years. % of lost to follow-up is not mentioned.
Fazal et al 2013 [11]	+	+	+	+	+	+	+	NA	7	Groups are comparable. % of lost to follow-up is not mentioned.
Rordorf et al 2013 [12]	+	+	+	+	+	+	+	NA	7	Groups are comparable. % of lost to follow-up is not mentioned.
Verlato et al 2013 [13]	+	+	+	+	+	+	-	NA	6	Groups are comparable. Mean follow-up duration is < 3 years. % of lost to follow-up is not mentioned.
Liu et al 2014 [14]	+	+	+	+	+	+	+	NA	7	Groups are comparable. % of lost to follow-up is not mentioned
Vollman et al 2014 [15]	+	+	+	+	-	+	+	+	7	Baseline differences among the two different lead families. % of lost to follow-up is mentioned and not likely to induce bias.
Yanagisawa et al 2014 [16]	+	+	+	+	+	+	+	NA	7	Groups are comparable. % of lost to follow-up is not mentioned
Kramer et al 2015 [17]	+	+	+	+	NA	+	+	NA	6	No comparison of baseline variables among different lead families. % of lost to follow-up is mentioned (>5%).

Legend: NA – not available.

Table S-2 – Head-to-head comparison of the 5 lead families: Sensitivity analysis excluding studies featuring mechanical failure as part of the combined endpoint.

Durata	Endotak Reliance	Fidelis	Sprint Quattro	Riata
**Durata**	E vs. D: 0.05<br>${}_{95\%}$CI -0.83,0.93<br>P=0.91; I^2=83%	D vs. F: -3.19<br>${}_{95\%}$CI -6.08,-0.30<br>P=0.03; I^2=86%	Q vs. D: -0.25<br>${}_{95\%}$CI -0.75,0.24<br>P=0.31; I^2=55%	D vs. R: -1.64<br>${}_{95\%}$CI -3.20,-0.09<br>P=0.04; I^2=76%
	**Endotak Reliance**	E vs. F: -2.18<br>${}_{95\%}$CI -2.63,-1.73<br>P<0.001; I^2=20%	E vs. Q: 0.01<br>${}_{95\%}$CI -0.20,0.22<br>P=0.91; I^2=44%	E vs. R: -0.66<br>${}_{95\%}$CI -1.26,-0.07<br>P=0.03; I^2=67%
		**Fidelis**	Q vs. F: -2.18<br>${}_{95\%}$CI -2.59,-1.78<br>P<0.001; I^2=57%	F vs. R: -1.38<br>${}_{95\%}$CI -1.65,-1.11<br>P<0.001; I^2=0%
			**Sprint Quattro**	Q vs. R: -0.82<br>${}_{95\%}$CI -1.42,-0.21<br>P=0.008; I^2=80%
				**Riata**

Legend: CI – confidence interval; D – Durata; E – Endotak Reliance; F – Sprint Fidelis; Q – Sprint Quattro; R – Riata.

References

1. Kleemann T, Becker T, Doenges K, Vater M, Senges J, Schneider S, Saggau W, Weisse U, Seidl K. Annual rate of transvenous defibrillation lead defects in implantable cardioverter-defibrillators over a period of >10 years. *Circulation.* 2007;115:2474-80.

2. Eckstein J, Koller MT, Zabel M, Kalusche D, Schaer BA, Osswald S, Sticherling C. Necessity for surgical revision of defibrillator leads implanted long-term: causes and management. *Circulation.* 2008;117:2727-33.

3. Hauser RG, Maron BJ, Marine JE, Lampert R, Kadish AH, Winters SL, Scher DL, Biria M, Kalia A. Safety and efficacy of transvenous high-voltage implantable cardioverter-defibrillator leads in high-risk hypertrophic cardiomyopathy patients. *Heart Rhythm.* 2008;5:1517-22.

4. Borleffs CJ, van Erven L, van Bommel RJ, van der Velde ET, van der Wall EE, Bax JJ, Rosendaal FR, Schalij MJ. Risk of failure of transvenous implantable cardioverter-defibrillator leads. *Circ Arrhythm Electrophysiol.* 2009;2:411-6.

5. Hauser RG, Hayes DL. Increasing hazard of Sprint Fidelis implantable cardioverter-defibrillator lead failure. *Heart Rhythm.* 2009;6:605-10.

6. Morrison TB, Friedman PA, Kallinen LM, Hodge DO, Crusan D, Kumar K, Hayes DL, Rea RF, Hauser RG. Impact of implanted recalled sprint Fidelis lead on patient mortality. *J Am Coll Cardiol.* 2011;58:278-83.

7. Parkash R, Thibault B, Sterns L, Sapp J, Krahn A, Talajic M, Luce M, Yetisir E, Theoret-Patrick P, Wells G, Tang A. Sprint Fidelis lead fractures in patients with cardiac resynchronization therapy devices: insight from the Resynchronization/Defibrillation for Ambulatory Heart Failure (RAFT) study. *Circulation.* 2012;126:2928-34.

8. Sung RK, Massie BM, Varosy PD, Moore H, Rumsfeld J, Lee BK, Keung E. Long-term electrical survival analysis of Riata and Riata ST silicone leads: National Veterans Affairs experience. *Heart Rhythm.* 2012;9:1954-61.

9. Abdelhadi RH, Saba SF, Ellis CR, Mason PK, Kramer DB, Friedman PA, Gura MT, DiMarco JP, Mugglin AS, Reynolds MR, Bazaz RR, Retel LK, Hayes DL, Hauser RG. Independent multicenter study of Riata and Riata ST implantable cardioverter-defibrillator leads. *Heart Rhythm.* 2013;10:361-5.

10. Ellenbogen KA, Gunderson BD, Stromberg KD, Swerdlow CD. Performance of Lead Integrity Alert to assist in the clinical diagnosis of implantable cardioverter defibrillator lead failures: analysis of different implantable cardioverter defibrillator leads. *Circ Arrhythm Electrophysiol.* 2013;6:1169-77.

11. Fazal IA, Shepherd EJ, Tynan M, Plummer CJ, McComb JM. Comparison of Sprint Fidelis and Riata defibrillator lead failure rates. *Int J Cardiol.* 2013;168:848-52.

12. Rordorf R, Poggio L, Savastano S, Vicentini A, Petracci B, Chieffo E, Klersy C, Landolina M. Failure of implantable cardioverter-defibrillator leads: a matter of lead size? *Heart Rhythm.* 2013;10:184-90.

13. Verlato R, Facchin D, Catanzariti D, Molon G, Zanotto G, Morani G, Brieda M, Zanon F, Delise P, Leoni L, Comisso J, Campo C. Clinical outcomes in patients with implantable cardioverter defibrillators and Sprint Fidelis leads. *Heart.* 2013;99:799-804.

14. Liu J, Brumberg G, Rattan R, Patel D, Adelstein E, Jain S, Saba S. Longitudinal follow-up of implantable cardioverter defibrillator leads. *Am J Cardiol.* 2014;113:103-6.

15. Vollmann D, Woronowicz S, Kmiec L, Jung K, Zenker D, Seegers J, Sossalla S, Dorenkamp M, Sohns C, Lüthje L, Hasenfuss G, Zabel M. Passive-fixation lead failure rates and long-term patient mortality in subjects implanted with Sprint Fidelis electrodes. *Europace.* 2014;16:258-64.

16. Yanagisawa S, Inden Y, Shimano M, Yoshida N, Ichiyanagi H, Fujita M, Ohguchi S, Ishikawa S, Kato H, Okumura S, Miyoshi A, Nagao T, Yamamoto T, Hirai M, Murohara T. Clinical outcome of implantable cardioverter defibrillators with recalled and non-recalled leads in Japanese patients. Increased failure rate of the Sprint Fidelis lead. *Circ J.* 2014;78:353-9.

17. Kramer DB, Hatfield LA, McGriff D, Ellis CR, Gura MT, Samuel M, Retel LK, Hauser RG. Transvenous Implantable Cardioverter-defibrillator Lead Reliability: Implications for Post-Market Surveillance. *J Am Heart Assoc*. 2015; 4:e001672 doi: 10.1161/JAHA.114.001672
